# Hierarchical interactions between Fnr orthologs allows fine-tuning of transcription in response to oxygen in *Herbaspirillum seropedicae*

**DOI:** 10.1093/nar/gky142

**Published:** 2018-02-26

**Authors:** Marcelo Bueno Batista, Govind Chandra, Rose Adele Monteiro, Emanuel Maltempi de Souza, Ray Dixon

**Affiliations:** 1 Department of Molecular Microbiology, John Innes Centre, Colney Lane, Norwich NR4 7UH, UK; 2 Department of Biochemistry and Molecular Biology, Universidade Federal do Parana, P.O. Box 19046, Curitiba, PR 81531-990, Brazil

## Abstract

Bacteria adjust the composition of their electron transport chain (ETC) to efficiently adapt to oxygen gradients. This involves differential expression of various ETC components to optimize energy generation. In *Herbaspirillum seropedicae*, reprogramming of gene expression in response to oxygen availability is controlled at the transcriptional level by three Fnr orthologs. Here, we characterised Fnr regulons using a combination of RNA-Seq and ChIP-Seq analysis. We found that Fnr1 and Fnr3 directly regulate discrete groups of promoters (Groups I and II, respectively), and that a third group (Group III) is co-regulated by both transcription factors. Comparison of DNA binding motifs between the three promoter groups suggests Group III promoters are potentially co-activated by Fnr3–Fnr1 heterodimers. Specific interaction between Fnr1 and Fnr3, detected in two-hybrid assays, was dependent on conserved residues in their dimerization interfaces, indicative of heterodimer formation *in vivo*. The requirements for co-activation of the *fnr1* promoter, belonging to Group III, suggest either sequential activation by Fnr3 and Fnr1 homodimers or the involvement of Fnr3–Fnr1 heterodimers. Analysis of Fnr proteins with swapped activation domains provides evidence that co-activation by Fnr1 and Fnr3 at Group III promoters optimises interactions with RNA polymerase to fine-tune transcription in response to prevailing oxygen concentrations.

## INTRODUCTION

The ability to tightly control transcription is an essential trait for bacterial fitness (1,2). In the rhizosphere, oxygen gradients may be important determinants that shape the bacterial community (3,4). The ability to rapidly adapt to varying oxygen conditions may be essential for successful rhizosphere colonization and the subsequent establishment of beneficial plant-bacteria interactions (5). *Herbaspirillum seropedicae* is a nitrogen fixing soil bacterium, able to colonize internal tissues of plants and stimulate growth of important agricultural crops (6–8). As in the case of many other bacteria, *H. seropedicae* takes advantage of a branched respiratory chain to cope with fluctuating oxygen concentrations (9,10). Reconfiguration of the respiratory chain in response to shifts in oxygen levels in *H. seropedicae* is controlled by three different orthologs of the global transcriptional regulator Fnr (named Fnr1, Fnr2 and Fnr3) (11). Fnr is a widespread transcriptional regulator in bacteria, which functions to reprogram gene expression in response to the transition from aerobic to anaerobic or microaerophilic growth conditions (12–14). Under conditions of oxygen deprivation, *Escherichia coli* Fnr binds one [4Fe–4S]^2+^ cluster per subunit, promoting conformational changes that facilitate protein dimerization and consequently DNA binding, resulting in transcriptional regulation at various promoters (15–18). Some organisms express multiple Fnr proteins that retain the cysteine residues required for [4Fe–4S]^2+^ cluster coordination, suggesting that each protein may have evolved to fulfil distinct oxygen responsive roles. Although several other bacteria, including *Burkholderia cenocepacia* (19), *Pseudomonas putida* (20) *Cupriavidus metalidurans* CH34 (21) and *Ralstonia eutropha* H16 (22) possess multiple *fnr* genes, the rationale for such redundancy is not completely understood. Potentially each transcriptional regulator may target different sub-sets of genes and have differential sensitivity towards oxygen, so that a hierarchy of oxygen-dependent transcriptional regulation is achieved.

To systematically address the regulatory role of two of these Fnr paralogs in *H. seropedicae*, we have performed RNA-Seq transcriptional profiling of single *fnr* deletion mutants in combination with ChIP-Seq to identify binding sites of each Fnr protein in the genome. We found that Fnr1 and Fnr3 specifically regulate discrete classes of genes (Groups I and II, respectively) but there is a third class of promoters (Group III) that are co-regulated by both transcription factors, with putative hybrid DNA binding sites, apparently comprising Fnr1 and Fnr3-specific half-sites. Using bacterial two-hybrid assays we found evidence for Fnr homodimer formation under anaerobic conditions and also for the interaction between Fnr1 and Fnr3, dependent upon specific side-chains in their dimerization helices, indicative of heterodimer formation *in vivo*. To further characterise Group III promoters, we have manipulated the Fnr DNA target sequence of a representative promoter and have analysed transcriptional activation by engineered Fnr proteins in which their respective activating domains have been swapped. Our data indicate that co-activation by Fnr1 and Fnr3 at Group III promoters provides fine-tuning of transcription and more efficient adaptation to prevailing oxygen levels.

## MATERIALS AND METHODS

### Bacterial strains and growth conditions

The bacterial strains used in this study are listed in Supplementary Table S1. *Escherichia coli* strains were grown at 37°C in LB medium (23) unless stated otherwise. *H.seropedicae* strains were grown at 30°C in NFbHP-Malate medium (24) supplemented with 20 mM of NH_4_Cl. The antibiotics used were ampicillin (250 μg ml^−1^ for *E. coli*), streptomycin (80 μg ml^−1^), nalidixic acid (5 μg ml^−1^), tetracycline (10 μg ml^−1^), kanamycin (50 μg ml^−1^ for *E. coli* and 500 μg ml^−1^ for *H. seropedicae*) and gentamycin (50 μg ml^−1^ for *E. coli* and 500 μg ml^−1^ for *H. seropedicae*). To evaluate the adaptation of *H. seropedicae* SmR1 to low oxygen levels we developed an oxygen switch protocol, in which the *H. seropedicae* strains were grown at 350 rpm (high aeration) until an O.D_600_ of ∼0.35 was reached and then cultures were switched to 120 rpm (low aeration) for 30 min. The activation of the Fnr1 protein after the high to low oxygen switch was validated by checking the activity of the *fixN* promoter before and after switch (Supplementary Figure S1).

### Recombinant DNA work

The plasmids and oligonucleotides used in this study are listed in Supplementary Tables S1 and S2, respectively. General molecular biology techniques such as PCR, DNA restriction and cloning, were performed according to established protocols (23). Restriction enzymes were provided by New England Biolabs or Thermo Scientific, while the high-fidelity DNA polymerase used for PCR was provided by Thermo Scientific. DNA purification was performed using commercially available kits provided by Macherey-Nagel or Qiagen. Sanger DNA sequencing was conducted by Eurofins MWG Operon while the oligonucleotide synthesis was conducted either by Eurofins MWG Operon or Integrated DNA Technologies. All plasmids and primers used in this study are listed in Supplementary Tables S1 and S2, respectively.

### Construction of *H. seropedicae* mutants

To avoid any concerns that might arise from the use of partial *in frame* deletions in the *fnr* genes used in a previous study (11), we constructed new *fnr* deletion mutants in which the coding sequence was almost completely removed. Only ∼30 bp from the 5′ and 3′ ends of each *fnr* each were retained to avoid polar effects on downstream genes and to facilitate mutant genotype verification by using internal primers. The peptide expressed from the remaining junction of the 5′ and 3′ ends of each *fnr* gene does not code for any structured domain characteristic of the Fnr protein. To construct the new *fnr* mutants, we generated deletions by overlapping PCR (25) using the primers described in the Supplementary Table S2 and the plasmids pMBB1D, pMBB2D and pMBB3D (Supplementary Table S1) as templates for *fnr1, fnr2* and *fnr3* genes, respectively. The products obtained from the overlapping PCR, were then digested with *Bam*HI and *Hind*III and cloned into pK18mobSacB vector (26), to generate the suicide plasmids pMB1231 (*fnr1* deletion), pMB1232 (*fnr2* deletion) and pMB1233 (*fnr3* deletion). To generate C-terminal 3xFlag tagged Fnr proteins, we first cloned the coding regions of the *fnr1* and *fnr3* genes into BamHI/XhoI sites of a pUC57-derived vector containing the sequence encoding the 3xFlag (pMB1300 synthesized by GenScript Corporation). Subsequently, a fragment of approximately 1.0 Kb downstream of each cognate *fnr* gene was cloned as a HindIII/XmaI fragment including the 3xFlag tag sequence to generate the plasmids pMB1301 and pMB1303. The *fnr3* construct in pMB1303 was then sub cloned as a M13F/M13R PCR product into pJET1.2/blunt to generate pMB1304. Finally the constructs on pMB1301 and pMB1304 were subcloned as BamHI/XmaI and XbaI/NotI respectively, into the pJQ200SK vector (26) to generate the suicide plasmids pMB1305 (*fnr1* tagging), and pMB1307 (*fnr3* tagging).

We used a similar approach to that described above for the generation of C-terminal 3xFlag tagged Fnr proteins, and to engineer genes expressing modified versions of Fnr1 and Fnr3 in which activating region 3 (AR3) was swapped. To facilitate immune detection of the swapped proteins we also added a C-terminal 3xFlag to these swapped proteins. The altered genes were generated by overlapping PCR using the primers listed in Supplementary Table S2 and cloned into *EcoR*V site of pBlueScript KS II+. The *fnr1^AR3→3-3xFlag^* gene was cloned into pBlueScript KS II+ generating the plasmid pMB1601. Subsequently, the BamHI/HindIII fragment of pMB1601 was cloned into pMB1301 to generate the plasmid pMB1609. Then, the BamHI/XmaI fragment from pMB1609 was cloned into pK18mobSacB to generate the suicide vector pMB1615 (*fnr1^AR3→3-3xFlag^*).

The *fnr3^AR3→1–3xFlag^*was also cloned into EcoRV site of pBlueScript KS II+ to yield the plasmid pMB1604. The downstream region of the *fnr3* gene (from pMB1304) was cloned as a *Hind*III/*Xho*I fragment into pMB1604 (*fnr3^AR3→1–3xFlag^*) linearized with HindIII/SalI to yield the plasmid pMB1612. Finally, the EcoRI/SmaI fragment from pMB1612 was cloned into pK18mobSacB (linearized with the same enzymes) to generate pMB1618 (*fnr3^AR3→1–3xFlag^*).

The suicide plasmids generated for gene replacements (to construct deletions, 3xFlag tagged or domain swap strains) were then transferred to *H. seropedicae* SmR1 by conjugation using *E. coli* S17.1 as a donor according to a previously described protocol (11). Single crossover strains for the deletion and tagging strategies were selected with kanamycin (pK18mobSacB derived plasmids) and gentamycin (pJQ200SK derived plasmids) resistance, respectively. Double crossover strains were counter-selected on 5% sucrose plates and then tested for specific antibiotic sensitivity. The final candidate mutant strains, sensitive to either kanamycin or gentamycin and resistant to sucrose, were genotyped by PCR using primers indicated in Supplementary Table S2. The integrity of the variant genes was checked by Sanger sequencing of PCR products obtained from candidate mutant strains.

### Construction of transcriptional fusions and BACTH plasmids

To study the expression profile of the *fnr1* gene in different *fnr* deletion backgrounds we constructed a transcriptional fusion named *pfnr1::lacZ*. Firstly, a DNA fragment corresponding to the *fnr1* promoter was generated by PCR and then cloned into the *Pst*I/*Bgl*II sites of pPW452 to generate the plasmid pMB1201. Another transcriptional fusion, containing a promoter with an altered Fnr binding site (pfnr1*::lacZ), was generated by overlapping PCR and cloned into EcoRV site of pBlueScript KS II + to generate pMB1607. The PstI/BglII fragment from pMB1607 was then subcloned into pPW452 to yield pMB1608 (pfnr1*::lacZ).

To construct the plasmids for the bacterial two hybrid analysis (BACTH) (27,28), DNA fragments encoding the protein fragment of interest flanked by a 5′ BamHI site and a 3′ KpnI site were generated by PCR. The fragments corresponding to *fnr1, fnr2* and *fnr3* genes were cloned into different BACTH vectors, linearized with the same enzymes, to create a range of plasmids expressing all possible combinations of Fnr proteins fused to *cyaA*-T25 or to *cyaA-*T18 at either their N- or C-terminal ends. The BACTH vectors carrying *fnr* genes with specific point mutations in the region coding for the dimerization helix were constructed in the same fashion, except that the desired mutations were introduced by overlapping PCR before cloning the mutated gene into BamHI and KpnI sites of the BACTH vectors. The complete list of plasmids obtained with their descriptions is presented in Supplementary Table S1.

### β-Galactosidase assays

β-Galactosidase assays were performed as described previously (11,29,30). To reach oxygen limiting conditions for assays using the *E. coli* BTH101 (bacterial two hybrid reporter) strain, we used 6 ml screw cap bijou universals filled to the top with LB medium supplemented with 1% glucose as described elsewhere (31). The initial O.D._600 nm_ was normalized to 0.2 and the strains were incubated at 30°C under 250 rpm overnight. Activity assays using *H. seropedicae* cultures were performed using cells grown under the oxygen switch protocol described above.

### Western blotting

Protein extracts for western blotting were prepared using *H. seropedicae* cultures prepared under the oxygen switch protocol described above, except that cultures aliquots were taken at different time points, as stated in the Figure legends. Cells were collected by centrifugation (6500 x g, 4°C, 5 minutes), resuspended to 1/10th of the initial volume in lysis buffer (10 mM Tris–Cl, pH 8.0, 50 mM NaCl, plus 1× protease cocktail inhibitor Roche #11836170001) and disrupted by sonication. The lysate was clarified by centrifugation (17 000 x g, 4°C, 5 min), protein quantified by using the Bio-Rad Protein Assay (#500-0002) and subsequently used for western blotting using the automated Simple Western™ system (Protein Simple©) according to previously established protocols (32,33). The capillaries were loaded with 0.3–0.5 μg of crude protein extract and the primary antibody (ANTI-FLAG^®^—Sigma #7425) dilution used was 1/500.

### RNA purification and high throughput sequencing (RNA- Seq)

*H. seropedicae* SmR1 (wild type), Δ1 (Δ*fnr1*), and Δ3 (Δ*fnr3*) strains were grown in 250 ml Erlenmeyer flasks containing 50 ml of culture medium at ‘high’ aeration or switched from ‘high’ to ‘low’ conditions as described above. After incubation, 30 ml of RNA Later™ (20 mM EDTA, 25 mM sodium citrate, 70 g ammonium sulphate/100 ml solution, pH 5.2) solution was added to 50 ml cultures, for RNA stabilization, and then split into falcon tubes containing 40 ml each. Cells were collected by centrifugation (6500 × g, 4°C, 5 min) and resuspended to a final volume of 200 μl with 10 mM Trizma^®^ (Sigma# T-2694) prepared in RNase free water. The cells were then mixed with 700 μl of RLT Buffer (Qiagen Rneasy Mini Kit #74104) containing 1% of β-mercaptoethanol and added to lysing tubes containing zirconia and silica/glass beads in the proportion of 2:1 (Thistle Scientific Ltd). Lysis was carried out with 3 pulses (speed 6.5 with 30 seconds on/1.5 min off) using the Thermo Savant FastPrep 120 Cell Disrupter System. Beads and cellular debris were collected by centrifugation (17 000 × g, 4°C, 5 min). The supernatant (900 μl) was transferred to a new RNase free tube and 450 μl of ethanol (Sigma #459844) was then added. The samples were applied to the RNeasy columns (Qiagen RNeasy Mini Kit #74104) and total RNA was recovered after *on column* DNAse treatment with the Qiagen RNase-Free DNase set (#79254) following the manufacturer's instructions. The quality of purified RNA was accessed by electrophoresis in a 1% agarose gel. RNA was treated with Turbo DNase (Ambion#AM1907) following the manufacturer's instructions and further purified with Qiagen RNeasy columns to avoid carryover of divalent cations. rRNA depletion was performed with Ribo-Zero Gram-negative Bacteria kit (Epicentre#MRZGN126) using 4 μg of total RNA as recommended by manufacturer. The mRNA enriched samples were sent for library construction and sequencing by The Earlham Institute (formerly known as The Genome Analysis Centre - TGAC), Norwich Research Park, Norwich, United Kingdom.

### Chromatin immunoprecipitation followed by high throughput sequencing (ChIP-Seq)

*H. seropedicae* SmR1 (wild type), Fnr1^−3xFlag^ (*fnr1::[Leu-Glu]-3XFlag*) and Fnr3^−3xFlag^ (*fnr3::[Leu-Glu]-3XFlag*) were grown under the same conditions described for the RNA-Seq. Immediately after the oxygen switch, cells were subjected to cross linking for 25 min with formaldehyde (Sigma#F8775) to a final concentration of 1% (vol/vol). Cross linking was quenched by incubating cells on ice for 5 min with 125 mM of glycine. Cells were collected by centrifugation and washed twice in 20 ml of PBS buffer pH 7.4 (Sigma#P4417). The washed pellets were then resuspended in 1.0 ml of IP lysis buffer (10 mM Tris–Cl, pH 8.0, 50 mM NaCl), containing 1x protease cocktail inhibitor (Roche#11836170001). Lysis and DNA shearing were performed by sonication. The lysate obtained was clarified by centrifugation at 4°C, 17 000 × g for 5 min and 25 μl of the supernatant combined with 75 μl of TE buffer (10 mM Tris–Cl, pH 7.4, 1 mM EDTA) was treated with 1 μl of RNase (Sigma#R4642) and extracted once with phenol:chlorofrom:isoamyl alcohol (25:24:1) followed by chloroform extraction. DNA shearing was confirmed by 1% agarose gel electrophoresis. Fragments ranged from 100 to 500 bp, centered on 300 bp.

Subsequently, 525 μl of IP buffer (50 mM Tris–Cl, pH 8.0, 250 mM NaCl, 0.8% [vol/vol] Triton X-100), containing 1x protease cocktail inhibitor, were added to the lysates (975 μl) and samples were chilled on ice. 50 μl of each lysate was set aside for total-DNA extraction. The remaining portion of the lysate was added to 45 μl of EZview Red ANTI-FLAG M2 affinity gel (Sigma#F2426) prepared and washed with TBS buffer (Sigma#T5030) as described in the manufacturer's instructions. The cross-linked products were immuno precipitated by incubation for 20 hours on a rotating wheel at 4°C. The samples were then centrifuged for 1 min at 4°C at 4500 × g, and the pellets were washed once with 1.5 ml of IP buffer and then three times more with 1 ml of IP buffer. After washing, the affinity gel pellets and the 50 μl of lysate (set aside earlier) were incubated overnight at 65°C in 100 μl of IP elution buffer (50 mM Tris–Cl, pH 7.6, 10 mM EDTA, 1% SDS). Samples were then centrifuged at 16 000 × g for 5 min to remove the affinity gel. The supernatants were transferred to new tubes and the affinity gel was re-extracted with 50 μl of TE buffer followed by a further 5 min incubation at 65°C. Sample volumes were adjusted to 200 μl with TE buffer and incubated with 3 μl of 10 mg/ml of proteinase K (Roche# 03115879001) for 2 h at 55°C. The samples were extracted once with phenol:chloroform:isoamyl alcohol (25:24:1) and once with chloroform and further purified using Macherey-Nagel NucleoSpin^®^ columns (Catalog#740609.50). DNA was eluted in 50 μl of nuclease-free H_2_O and quantified by using the Nanodrop (Thermo Scientific).

The purified DNA was sequenced in an Illumina HiSeq at The Earlham Institute (formerly known as The Genome Analysis Centre-TGAC, Norwich Research Park, Norwich, United Kingdom). The TruSeq ChIP sample preparation kit from Illumina Inc. was used as reported previously (34).

### ChIP-Seq and RNA-Seq Data analysis

The reads in the fastq files received from the sequencing contractor were aligned to the *H. seropedicae* genome (GenBank accession number: NC_014323.1) using the bowtie2 software (35), which resulted in one SAM (.sam) file for each fastq file. All further operations for the ChIP-Seq analysis were carried out using a combination of Perl scripts dependent on the BioPerl toolkit (36) and R scripts essentially as described previously (34). Statistical significance of ChIP-Seq enriched peaks was determined by comparing the level of enrichment between the total and immunoprecipitated (IP) libraries with *P* < 0.0001 considering that differences between samples were distributed normally (34). The differential expression analyses for RNA-Seq was performed using edgeR (37). Differentially expressed genes had *P* <0.001 and 1 ≤ logFC ≤ –1.

### Determination of Fnr DNA-binding motifs

For identification of Fnr binding motifs, promoters with an associated ChIP-Seq peak that exhibited differential expression in the RNA-Seq datasets were selected and submitted to motif identification using a MEME search (38). Briefly, a sequence of 300 bp surrounding the centre of the ChIP-Seq peak (150 bp upstream and 150 bp downstream from the centre) was selected in a strand specific manner. A fasta file containing all sequences for a given regulatory category was generated and then submitted for MEME search using the on line submission platform (38). 39 promoter sequences were submitted for Group I promoters (Fnr1 motif search), while 23 and 18 promoter sequences were submitted for Group II and Group III promoters for the Fnr3 and Fnr3–Fnr1 motif searches, respectively. The sequences used for the MEME search as well as the list of motifs found are available in the Supplementary Files S1 and S2, respectively.

## RESULTS

### Influence of oxygen limitation on the transcriptome of *H. seropedicae*

To systematically address the role of the different Fnr proteins in transcription regulation we have established an oxygen switch protocol to study transcriptional changes directly or indirectly associated with these oxygen-sensitive regulatory proteins. To ensure an efficient transition from high oxygen levels to oxygen depletion, cells were grown to mid log phase (O.D._600 nm_ = 0.35) at high aeration rates (350 rpm) and then switched to oxygen-limiting conditions (120 rpm) for 30 min prior to RNA extraction. Under these conditions, the switch in oxygen availability has minimal impact on the growth rates of either the wild-type or the single *fnr* mutant strains in the short-term, whilst allowing the detection of Fnr-dependent promoter activation (Supplementary Figure S1). This methodology enabled comparison of transcript profiles among the different strains under ‘high aeration’ (350 rpm or control) and ‘low aeration’ (120 rpm or switch) conditions. Using cultures grown to equivalent optical densities under these conditions, we performed RNA-Seq analysis in combination with ChIP-Seq to address the specific regulons of both Fnr1 and Fnr3.We also carried out transcript profiling of the single *fnr2* deletion strain, but as few genes showed differential expression and we could not detect the expression of Fnr2 for ChIP-Seq analysis under these conditions, the function of Fnr2 was not studied further in this analysis.

An overview of the RNA-Seq data sets obtained in this study is shown in Supplementary Figure S2. The most highly induced transcripts in the wild type strain upon the switch from high to low aeration conditions include those encoding the structural components of the *cbb_3_*-type (*fixNOP*) terminal respiratory oxidase and the *ompW1* gene that encodes an outer membrane protein (shaded in blue in Supplementary Figure S2). Interestingly these genes are in the neighbourhood of the *fnr1* gene. Other notable highly-induced genes were *petABC* encoding the cytochrome *bc_1_*-complex (shaded in orange in Supplementary Figure S2) and *uspA1* coding for a universal stress protein (shaded in light red in Supplementary Figure S2). Both the *bc_1_* and *cbb_3_* complexes are likely to be essential targets for re-configuration of the cytochrome *c*-type electron transport chain, when oxygen is limiting, confirming previous transcriptome analysis of a triple *fnr* deletion strain of *H. seropedicae* (11).

**Figure 1.**
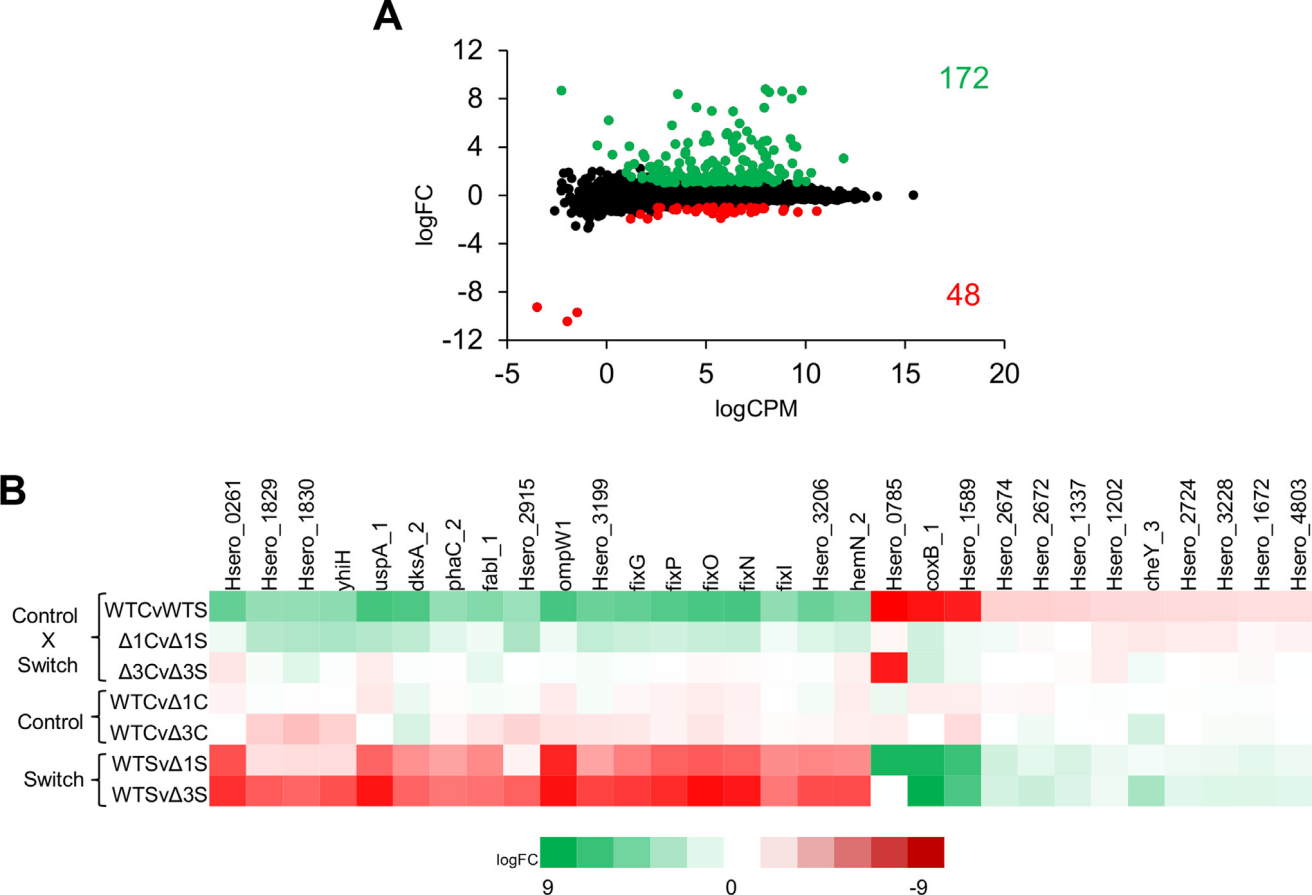
Transcriptional profiling reveals differential gene expression after the switch from high (control, C) to low (switch, S) oxygen conditions. (**A**) Plot showing the relationship between the average expression (logCPM) and fold-change (logFC) for the RNA-Seq library comparisons (control versus switch) in the wild type strain (WTCvWTS). Green dots indicate genes that were up regulated upon switch to low oxygen, while the red dots represent the genes that were down regulated (*P* < 0.001 and 1 ≤ logFC ≤ –1). (**B**) Heat map showing a subset of the most highly differentially expressed genes (up and down regulated) across the comparisons made between all transcript mappings. The abbreviations indicate the following: WT – wild type (SmR1); Δ1 – *fnr1* deletion; Δ3 – *fnr3* deletion; C – control (high aeration, 350 rpm); S – switch to low aeration (120 rpm). The differential expression comparisons are: WTCvWTS, wild type control (350 rpm) versus switch (120 rpm); Δ1CvΔ1S, *fnr1* deletion control versus switch; Δ3CvΔ3S, *fnr3* deletion control versus switch; WTCvΔ1C, wild type versus *fnr1* deletion under control conditions; WTCvΔ3C, wild type versus *fnr3* deletion under control conditions; WTSvΔ1S, wild type versus *fnr1* deletion under switch conditions; WTSvΔ3S, wild type versus *fnr3* deletion under switch conditions.

Comparison of the RNA-seq datasets of the wild type strain revealed that 220 genes were differentially regulated in response to the change in oxygen concentration. Of these, 172 genes were up regulated under low aeration, while 48 genes were down regulated (Figure 1A and Supplementary Table S3). A subset of the most highly differentially expressed genes, selected by comparison of datasets from cultures grown in high aeration (control, C) versus cultures switched to low aeration (switch, S), is shown in Figure 1B (see Figure 1B legend for description of library comparisons). Differential expression of genes in response to the switch to low aeration was abrogated in most cases in the *fnr3* deletion mutant (Figure 1B, compare Δ3CvΔ3S with WTCvWTS). In contrast, differential expression was only partially affected by the *fnr1* deletion (Figure 1B compare Δ1CvΔ1S with WTCvWTS). However, some genes were down regulated in the *fnr3* mutant even under conditions of high aeration (see WTCvΔ3C in Figure 1B), which was not observed with the *fnr1* mutant (see WTCvΔ1C), perhaps indicating that Fnr3 is less sensitive to oxygen than Fnr1. Finally, this analysis shows that most of the up-regulated genes in the WTCvWTS comparison were down-regulated in the WTSvΔ1S and WTSvΔ3S comparisons. Similarly, most of the down-regulated genes in WTCvWTS were up-regulated in WTSvΔ1S and WTSvΔ3S. Overall, this indicates that transcriptional regulation of most genes following the switch to low oxygen (WTCvWTS) requires both Fnr1 and Fnr3.

### RNA-Seq combined with ChIP-Seq unambiguously reveals the Fnr targets in *H. seropedicae*

**Figure 2.**
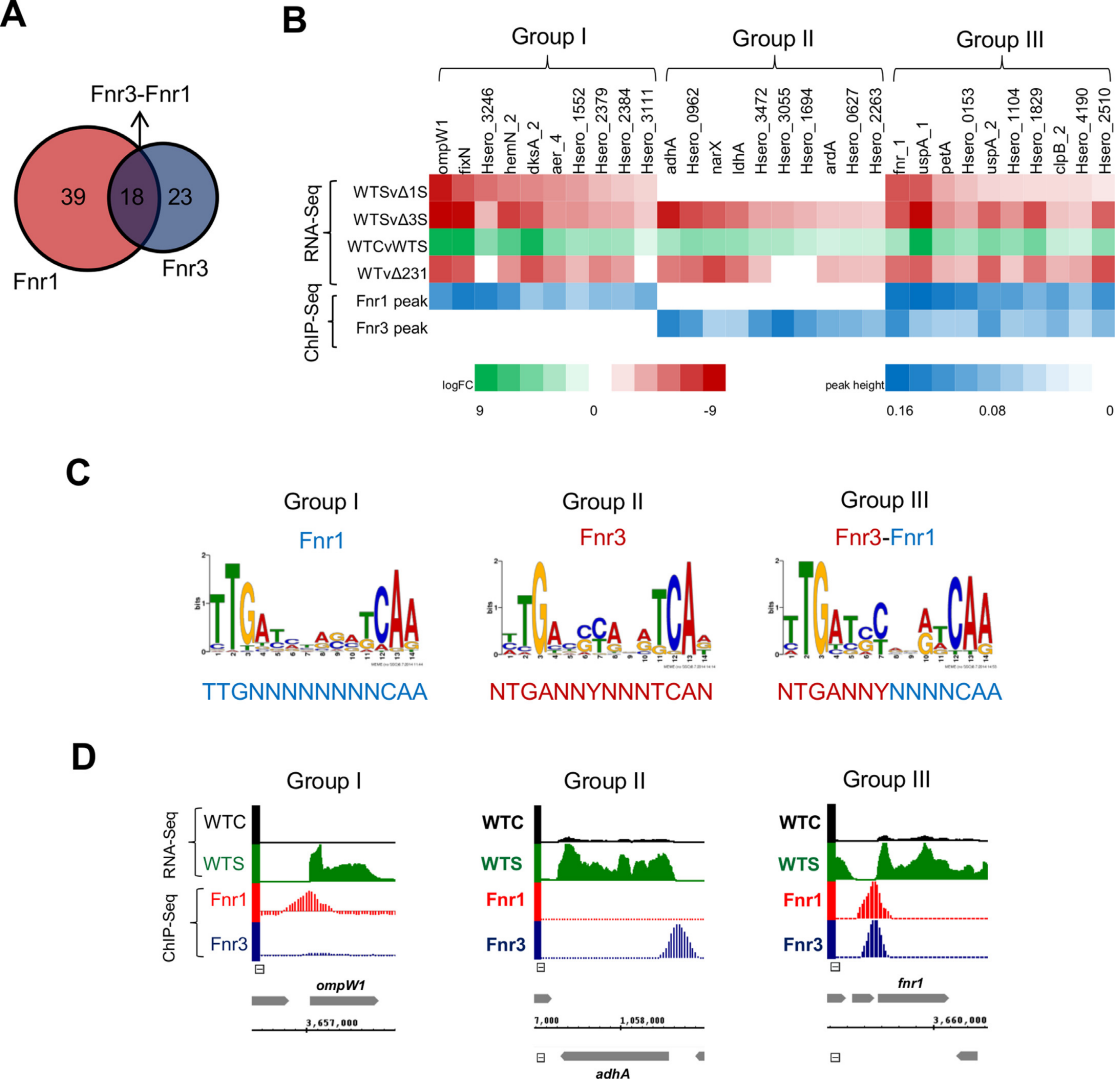
Correlation of RNA-Seq and ChIP-Seq reveals three groups of regulated promoters. (**A**) Overview of the number of specific targets identified for Fnr1 and Fnr3 identified by correlation of ChIP-Seq peaks with the transcriptional changes revealed by RNA-Seq. (**B**) Heat map showing the correlation between the differential expression revealed by the RNA-seq with the ChIP-Seq peak heights. For each regulatory group (Groups I, II and III) identified, a subset of the most highly differentially expressed genes associated with significant ChIP-Seq peak enrichment are shown. The differential expression comparisons are: Δ1CvΔ1S, *fnr1* deletion control versus switch; Δ3CvΔ3S, *fnr3* deletion control versus switch; WTCvWTS, wild type control (350 rpm) versus switch (120 rpm); WTvΔ231, wild type versus triple *fnr* deletion under 5% oxygen (11). (**C**) DNA-binding motifs identified by MEME for Groups I, II and III of regulated promoters. (**D**) Visualization of ChIP-Seq peaks in correlation with transcripts mapping to representative genes within each regulatory group. Abbreviations and colour coding for the RNA-Seq data are as follows: WTC, wild type under 350 rpm (black); WTS, wild type after switch to 120 rpm (green). The ChIP-Seq data for Fnr1–3xFlag and Fnr3–3xFlag strains is presented in red and blue, respectively.

To determine the direct promoter targets of Fnr protein, we correlated transcriptional changes observed in single *fnr1* and *fnr3* mutants with ChIP-Seq analysis using strains expressing C-terminally 3xFlag *fnr* alleles engineered into the *H. seropedicae* genome. The Flag tags did not influence the activity of Fnr proteins as judged by transcription activation of the *fixN* promoter, previously identified as a target for Fnr1 and Fnr3 (11) (Supplementary Figure S3). ChIP-seq data was obtained from cultures grown under limited oxygen availability using the same conditions used for the RNA-seq analysis. Using this approach, DNA-binding targets for the *H. seropedicae* Fnr proteins were unambiguously revealed and correlated with transcript profiles to determine the specific regulons of each protein. The Fnr1–3xFlag protein bound to 57 promoters with associated transcription changes among the different RNA-Seq datasets, while the Fnr3-3xFlag protein bound to 41 promoters. These promoters were classified in three different groups, designated as Group I, II and III. Group I comprises 39 promoters that were exclusively bound by Fnr1 (Supplementary Table S4 and Figure 2), while Group II includes 23 promoters exclusively bound by Fnr3 (Supplementary Table S5 and Figure 2). As *fnr1* expression is dependent upon Fnr3 (Supplementary Figure S4), all the genes in Group I were down regulated in both *fnr1* and *fnr3* mutants. In contrast, for Group II there was no detectable down regulation in the *fnr1* deletion strain, confirming that these promoters are exclusively regulated by Fnr3. Interestingly, we identified a third category of promoters (Group III), comprising 18 promoters that were bound by both the Fnr1 and Fnr3 proteins (Supplementary Table S6 and Figure 2). The co-immunoprecipitation of Fnr1 and Fnr3 to promoters in Group III indicates that these promoters are directly co-regulated by Fnr1 and Fnr3. Amongst Group I, we identified 5 target promoters that are repressed by Fnr1 under oxygen-limiting conditions (Supplementary Table S4), while for Group II, only 2 repressed promoters were identified (Supplementary Table S5). No repressed genes were found in Group III, suggesting that promoters in this category are subject only to activation (Supplementary Table S6). Altogether, these findings highlight the role of Fnr1 and Fnr3 as global dual regulators of transcription, with a major role in the activation of gene expression rather than in the repression.

A subset of the most relevant direct targets for each regulatory group is presented in Figure 2B. These promoters were selected based on the strength of Fnr binding, judged by the ChIP-Seq peak enrichments in combination with the level of differential expression in the RNA-Seq data. Regulated promoters in Group I, include those of the *ompW1* and the *fixNOP* operon, which are in the genomic neighbourhood of *fnr1* (Supplementary Figure S2 and Figure 2B). Other operons such as *hemN2-Hsero_3206-fixIS* and *dksAphaC2fabI-Hsero_2407* are directly regulated by Fnr1. Genes involved in the synthesis of polyhydroxybutyrate (PHB) including *phaC1*, which is essential for PHB accumulation (39,40) and an acetyl-CoA acetyltransferase encoded by *phbA2* (Supplementary Table S4) are also direct targets. The Fnr1 regulon also includes promoters of genes encoding proteins that may be related to the taxis response, including homologs of MCPs (Methyl Accepting Proteins), *aer_4* (Hsero_3072) and *tsr_6* (Hsero_3488).

Fnr3 directly activates genes in Group II, including those encoding alcohol dehydrogenase (*adhA*), d-lactate dehydrogenase *(ldhA)* and the *bd*-type quinol oxidase (*cydAB)*.The expression of genes related to the PHB metabolism may also be influenced by Fnr3, since the phasin protein encoded by Hsero_1639,which is the most abundant protein associated with PHB (39) is directly activated by this paralog. Fnr3 also directly targets the NarXL two component system (Figure 2 and Supplementary Table S5) which is important for the regulation of nitrate metabolism genes (41). In addition, a diverse group of transcription factors, including regulators of the TetR/AcrR (Hsero_0253), LysR (Hsero_1103), DeoR (Hsero_1024), GntR (Hsero_3146) and MerR (Hsero_1694 and Hsero_1710) families, are members of Group II (Supplementary Table S5). The functions of these transcription regulators remain to be elucidated, but potentially may allow *H. seropedicae* to integrate the oxygen status, via Fnr3 activity, with other regulatory signals.

Group III, which comprises promoters that are co-activated by Fnr1 and Fnr3, includes genes coding for important components of the electron transport chain and the stress response. These include cytochrome *c*553 (Hsero_0153), cytochrome *c*551/*c*552 (Hsero_1104), the *bc*_1_-complex (*petABC*) and two universal stress proteins (*uspA_1* and *uspA_2*). The latter are amongst the most highly expressed genes activated by the oxygen switch (Figure 2B and Supplementary Figure S2). It is important to note that the *fnr1* gene itself, falls within Group III (Figure 2 and Supplementary Table S6), requiring both Fnr1 and Fnr3 for full activation. This observation, together with the fact that expression of *fnr1* is dependent upon Fnr3, may indicate that once Fnr3 triggers the initial activation of *fnr1*, the co-activation mechanism ensures optimum expression levels and finely tuned regulation dependent upon oxygen levels (see Supplementary Figure S4 and Figure 5).

In addition to identifying direct targets for Fnr1 and Fnr3, correlation of the ChIP-Seq and RNA-Seq data also enabled us to unambiguously select subsets of promoters for prediction of Fnr binding motifs from each promoter class (Figure 2C). All promoters within each regulatory category had significant ChIP-Seq peaks (*P*-value < 1 × 10^−5^) and decreased RNA-Seq transcripts observed in the respective single deletion strain (Figure 2 and Supplementary Tables S3 to S6). Discrete binding motifs for each promoter class were identified using MEME searches (Supplementary Files S1 and S2). The Group I binding motif suggests that Fnr1 recognizes an inverted palindromic sequence, very similar to the canonical Fnr*-like* binding sites described for the Fnr and FixK2 proteins from *E. coli* and *Bradyrhizobium japonicum*, respectively (42). The Group II binding motif suggests Fnr3 recognizes a slightly different DNA-target sequence in which the flanking T residue at the 5′end of the motif (position 1) and the corresponding A residue at the 3′end (position 14) are not well conserved and there is a preference for C/G and C/T residues at positions 6 and 7 respectively. Interestingly, the Group III category, appears to be a hybrid sequence representing half-sites derived from both Group I and Group II motifs (Figure 2C). This may suggest that these promoters are co-regulated by Fnr1 and Fnr3. Since the upstream half-site resembles that of the Group II category (promoters bound by Fnr3-only), and the downstream half-site is more similar to the Group I category (promoters bound by Fnr1-only) the co-regulation of these promoters may occur through activation by Fnr3–Fnr1 heterodimers.

### The Fnr3 and Fnr1 proteins interact *in vivo*

**Figure 3.**
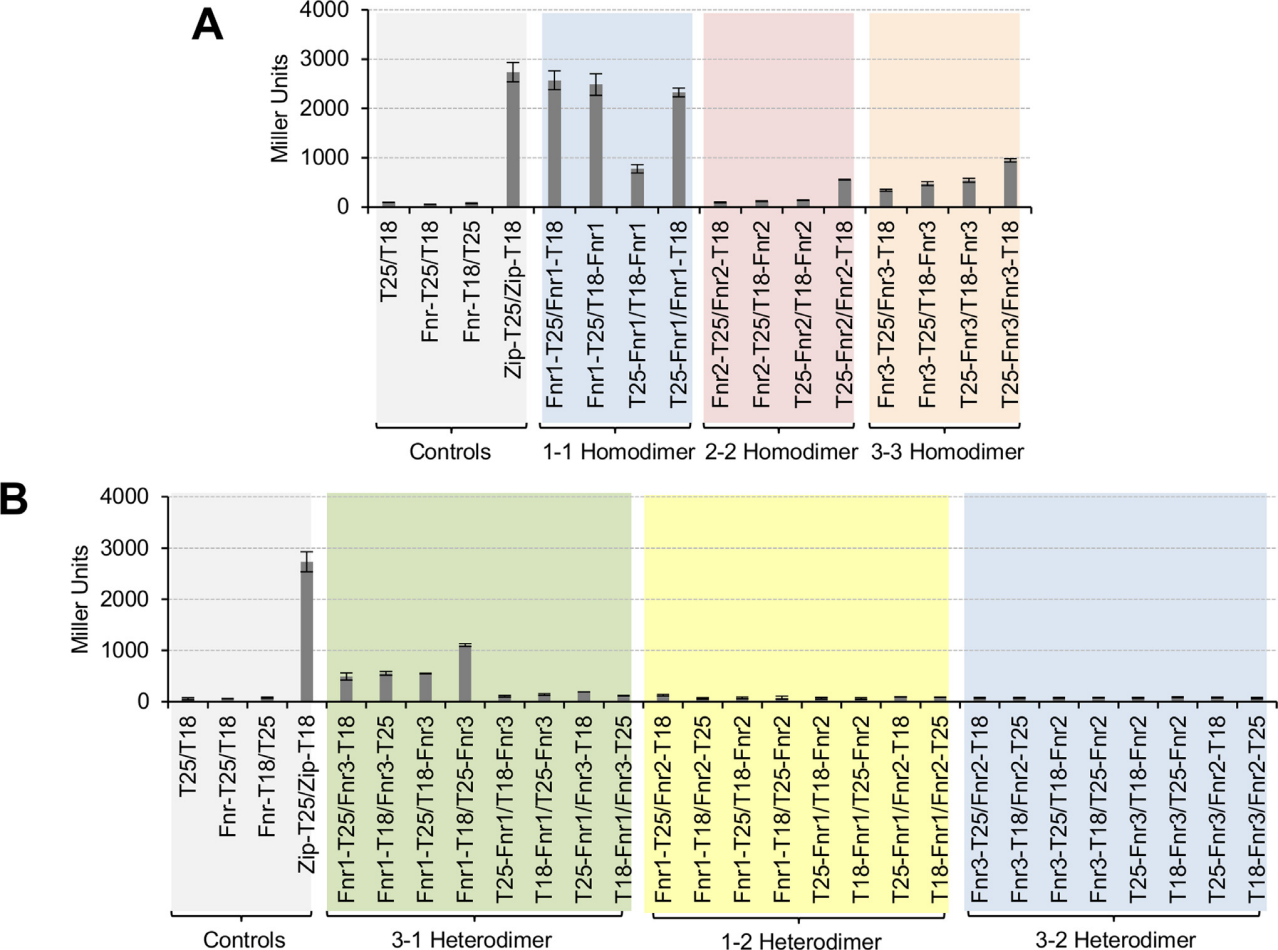
Heterodimers between Fnr1 and Fnr3 proteins are formed *in vivo*. (**A**) β-Galactosidase activity from the BACTH assay to test the homodimerization of Fnr1, Fnr2 and Fnr3. The interaction between Fnr proteins was tested using all possible combinations of fusion proteins. Negative controls: empty two-hybrid vectors (T25/T18); Fnr-T25 fusion protein combined with the empty vector carrying the T18 subunit (Fnr-T25/T18) and the Fnr-T18 fusion protein combined with the empty vector carrying the T25 subunit (Fnr-T18/T25). Fnr-T25/T18 and Fnr-T18/T25 denote that all three Fnr fusion proteins were tested for the unspecific interaction with either T18 or T25 fragments. The data shown is the average activity for all possible combinations. Positive control: we used the leucine zipper domain fusion proteins from the BACTH system (Zip-T25/Zip-T18). (**B**) β-galactosidase activity from the BACTH assay to test heterodimerization between different heterologous pairs of Fnr proteins (Fnr3–Fnr1, Fnr1–Fnr2 and Fnr3–Fnr2). For each Fnr pair, all eight possible pairwise combinations of fusion proteins were tested. The graph is shaded in different colours to facilitate identification of the different sets of interactions tested. β-galactosidase assays were performed using cultures grown under oxygen-limiting conditions. Error bars show standard deviation of three independent biological replicates carried out for each pairwise combination.

We used the bacterial two-hybrid system to evaluate interactions between the Fnr proteins *in vivo* in an *E. coli* background. To do this we constructed genes encoding fusions between Fnr1, Fnr2, and Fnr3 and the T-18 and T-25 adenylate cyclase subunits at both their N- and C-termini and then tested the ability of the different fusion proteins to interact with each other. To confirm that the interaction between *H. seropedicae* Fnr proteins can be studied using the bacterial two hybrid system as reported for *E. coli* Fnr (43), we tested the ability to detect homodimer formation by each Fnr protein using four different fusion protein combinations. Since oxidation of the [4Fe–4S]^2+^ cluster in Fnr results in inactivation of the protein through dimer to monomer conversion (44), we performed these experiments under limiting oxygen conditions. Homodimerization was detected with all fusion combinations for Fnr1 and Fnr3, even though the strength of the interaction between different fusion proteins was variable as judged by the levels of β-galactosidase activity (Figure 3A). Homodimerization of Fnr2 could only be detected when we used the T25-Fnr2 / Fnr2-T18 fusion combinations. This combination also gave the highest level of activity when measuring Fnr3 homodimerization. Notably all these interactions were undetectable when cultures were grown under aerobic conditions (data not shown), concomitant with the requirement for an intact [4Fe4S]^2+^ cluster for maintenance of the homodimer.

We next tested the ability of heterologous Fnr proteins (Fnr3–Fnr1, Fnr1–Fnr2 and Fnr3–Fnr2) to interact with each other, using all eight fusion protein combinations for each Fnr pair (Figure 3B). The only protein pair that gave rise to high β-galactosidase activity was Fnr3–Fnr1, suggesting that Fnr3 and Fnr1 can interact, driven by a heterodimer interface as predicted from the features of the Group III promoters.

**Figure 4.**
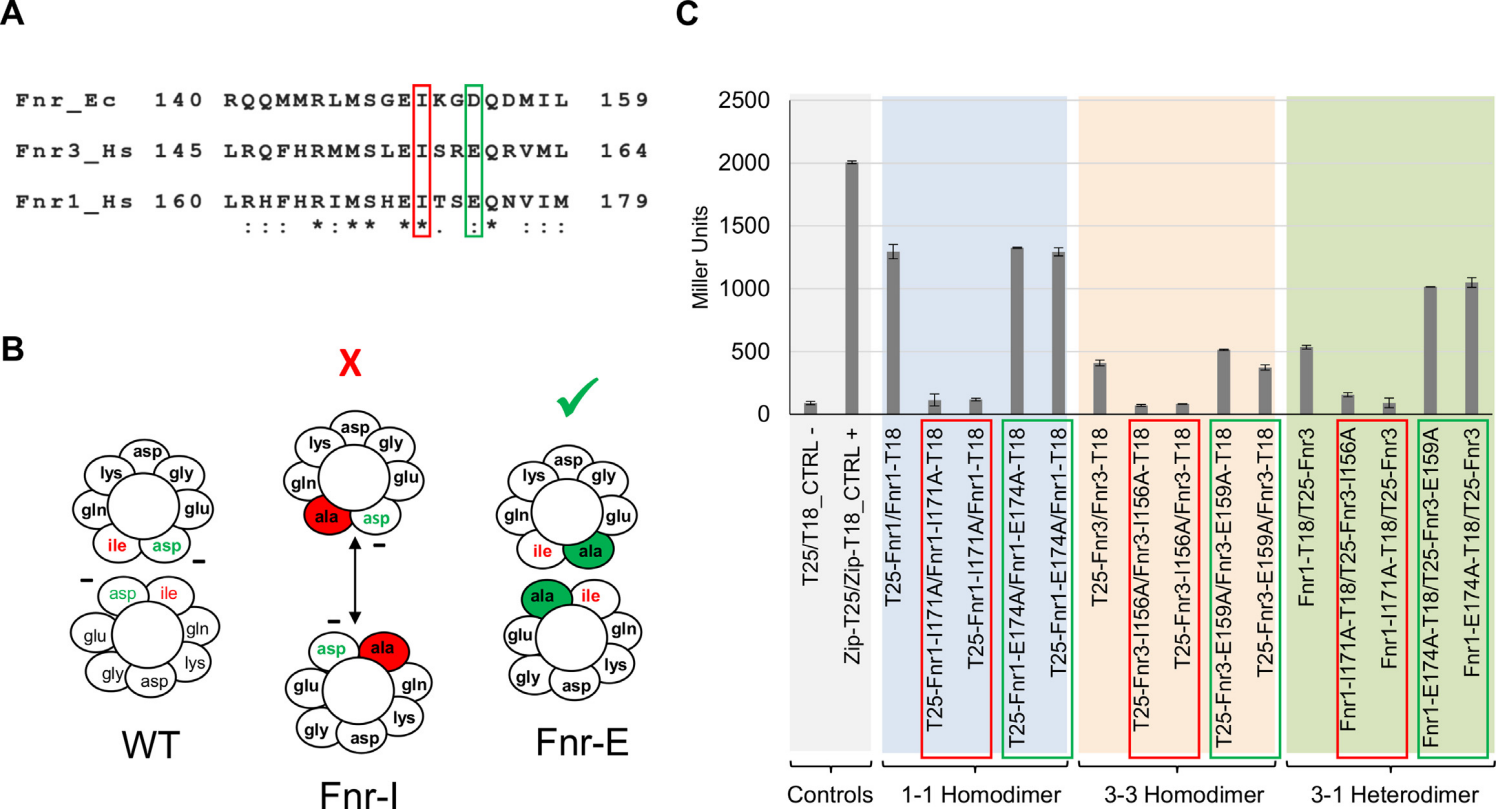
Interaction between Fnr1 and Fnr3 requires the dimerization helix. (**A**) Dimerization helix alignment from *E. coli* Fnr (Fnr_Ec) and *H. seropedicae* Fnr1 (Fnr1_Hs) and Fnr3 (Fnr3_Hs) proteins. Amino acid residues important for the interaction between subunits are highlighted in red and green. (**B**) Cross-section of the dimerization interface of wild type Fnr, FnrI151A and FnrD154A. Diagrams are based on the *E. coli* Fnr sequence (45,46). The charge of Asp154 is partially shielded from the interface by the presence of Ile151. Removal of the large hydrophobic residue at position 151 (Ile → Ala) allows the negative charge of Asp154 to become exposed in the interface and the repulsive interaction between the two Asp residues prevents dimerization. In the absence of the negatively charged residue at position 154 (Asp → Ala) dimerization is improved. Fnr-I indicates an Ile to Ala substitution whereas Fnr-E indicates a Glu to Ala change in the *H. seropedicae* Fnr proteins. (**C**) β-Galactosidase activity from the BACTH assays using Fnr1 and Fnr3 proteins carrying point mutations at positions relative to 151 and 154 (respective to the *E. coli* protein).The graph is shaded in different colours to facilitate identification of the different sets of interactions tested. β-galactosidase assays were performed using cultures grown under oxygen-limiting conditions. Error bars show the standard deviation of three independent biological replicates carried out for each pairwise combination.

To confirm that the dimerization helix is the unique surface of interaction between the monomeric units of Fnr1 and Fnr3, we made specific amino acid substitutions within the dimerization helix, to study the specificity of the interaction. Analysis of the *E. coli* Fnr protein dimerization helix has identified amino acid residues that are major players in the interactions that modulate Fnr dimerization (45–47) (Figure 4A and B). The large hydrophobic side chain of I151 in *E. coli* Fnr is believed to shield the negative charge from D154 to minimize the charge repulsion between two monomeric units to allow dimerization upon [4Fe–4S]^2+^ cluster binding. Alanine scanning mutagenesis of the dimerization helix from the *E. coli* Fnr (45), demonstrated that the I151A substitution completely abolishes dimerization, whilst the D154A substitution leads to a protein that is able to dimerize even in the absence of the [4Fe–4S]^2+^ cluster (45,46). By comparing the dimerization helix of *E. coli* Fnr protein with the helices from Fnr1 and Fnr3, we identified reciprocal amino acid residues in the *H. seropedicae* proteins (Figure 4A), and then evaluated the effect of the equivalent amino acid substitutions (I151A and D154A in *E. coli* Fnr) on both the homodimerization and heterodimerization (Figure 4).

As anticipated, our results demonstrate that Fnr1-I171A and Fnr3-I156A (equivalent to *E. coli* Fnr-I151A) are unable to form either homodimers or heterodimers as reported by the bacterial two-hybrid assays (Figure 4B). Additionally, we observed that the *H. seropedicae* Fnr substitutions, Fnr1-E174A and Fnr3-E159A (equivalent to *E. coli* Fnr-D154A) were not affected in their ability to form homodimers, but were found to have improved ability to form heterodimers under the conditions tested (Figure 4B). In all cases, only one mutant subunit was required to give rise to the observed changes in the interaction, as anticipated from the relatively weak hydrophobic interface (47). Since heterologous interactions with Fnr2 were not detected, and considering that dimerization is influenced by specific amino acid substitutions within the dimerization helix, the interaction between Fnr1 and Fnr3 is apparently highly specific *in vivo*.

### Group III promoter co-activation by Fnr1 and Fnr3

Co-activation of promoters by Fnr1 and Fnr3 could potentially be achieved by synergistic binding of these activators to tandem binding sites. However, we did not detect additional consensus binding sequences for Fnr, either upstream or downstream of the motif identified in promoters belonging to this group. The presence of overlapping Fnr1 and Fnr3 ChIP-seq peaks at Group III promoters suggests alternative models are feasible, including initial binding of Fnr3 homodimers to a single recognition site followed by protein exchange to enable binding and subsequent activation by Fnr1 homodimers. However, the nature of the motif identified for Group III promoters also suggests the possibility of regulation by Fnr3–Fnr1 heterodimers.

**Figure 5.**
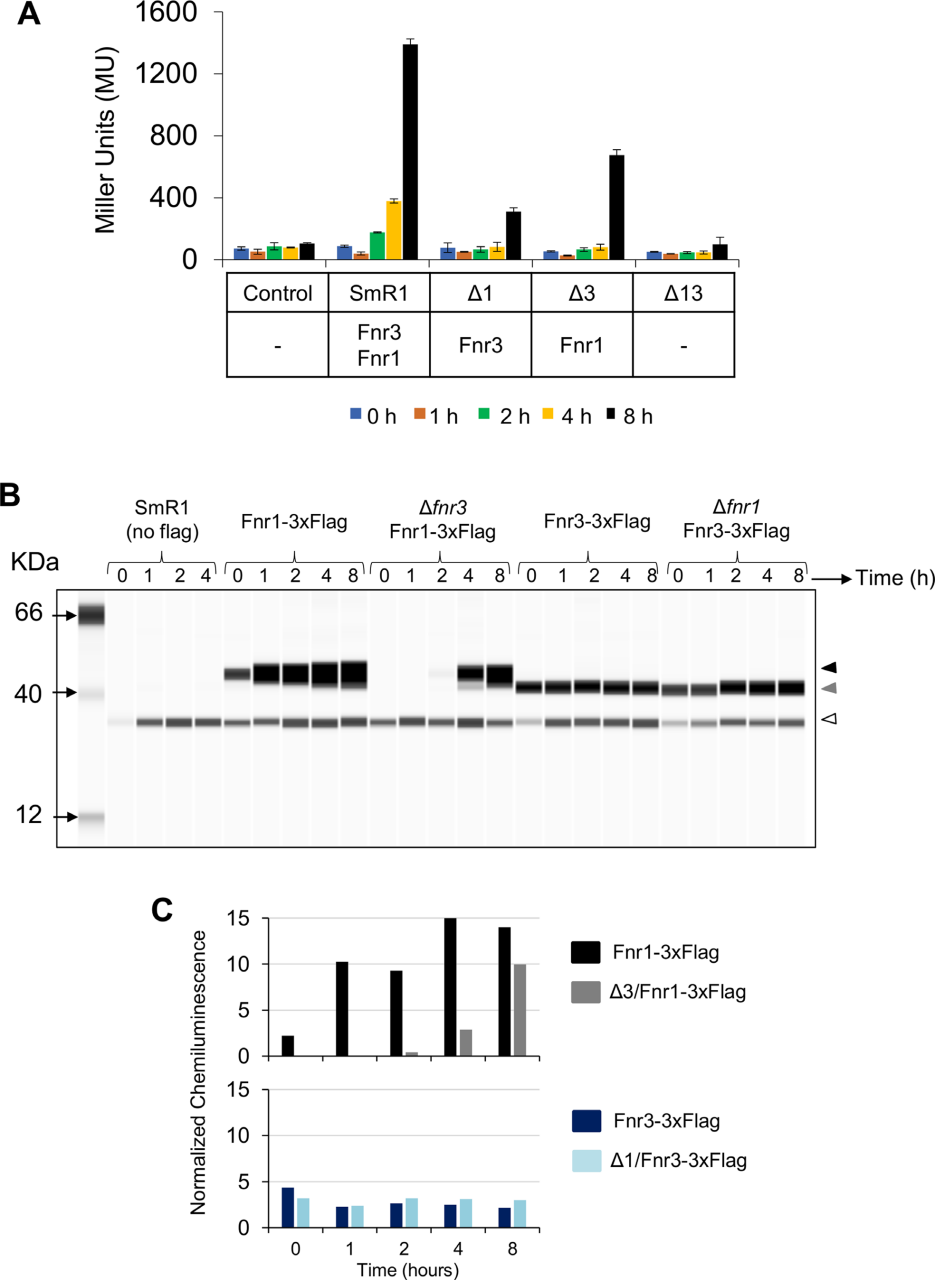
Co**-**activation of Group III promoters by Fnr1 and Fnr3. (**A**) Activity of the *pfnr1::lacZ* fusion in different *H.seropedicae* strains. Control, denotes the activity of a promoter less *lacZ* plasmid (pPW452) in the wild type strain of *H. seropedicae*. β-galactosidase activity was assayed as described in Materials and Methods using cultures incubated for zero (blue), 1 (brown), 2 (green), 4 (yellow), and 8 (black) hours respectively, after the switch from high to low oxygen. The *H. seropedicae* strain used, as well as the Fnr proteins expressed in these strains are indicated below the graph. The standard error is representative of three independent biological replicates. (**B**) Levels of Fnr1 and Fnr3 in the wild type and single deletion backgrounds were assessed using the Simple Wes system as described in Materials and Methods. Protein samples were taken under high aeration (time 0) and 1, 2, 4 and 8 h after switch from high to low aeration. The band of ∼35 kDa represents cross-reacting material present even in wild-type *H. seropedicae* lacking a 3X Flag tagged protein. The black arrowhead indicates Fnr1, the grey arrowhead indicates Fnr3, and the white arrowhead indicates the cross reacting protein. (**C**) The chemiluminescence signal from the digital western image in B was used to compare Fnr levels between wild type and single deletion *fnr* strains. The upper graph compares Fnr1 levels in the wild type (black) and in the single *fnr3* deletion (grey). The lower graph compares Fnr3 levels in the wild type (blue) and in the single *fnr1* deletion (light blue). The results are representative of two biological replicates.

To further characterise co-activation by Fnr1 and Fnr3 at Group III promoters, we analysed the expression profile of a *pfnr1::lacZ* transcriptional fusion, expressed on a plasmid in *H. seropedicae*. Fnr derivatives, expressed from their native locations in the *H. seropedicae* genome were constructed with 3xFlag epitopes fused to their C-termini to allow easy immunogenic verification. β-galactosidase assays were performed at various time points after the switch to low aeration and were correlated with the expression levels of Fnr1 and Fnr3 determined by quantitative western blotting of extracts prepared at each time point. The response of the *fnr1* promoter fusion in different *fnr* deletion backgrounds confirmed that full activation is only achieved when both *fnr1*and *fnr3* genes are present and no promoter activity was observed when both *fnr1* and *fnr3* were absent (Figure 5A and Supplementary Figure S5). Although the accumulated level of Fnr3 remained mostly unchanged in the wild-type strain during the time course, the level of Fnr1 increased by ∼5 fold one hour after the switch (resulting in an Fnr1:Fnr3 ratio of approximately 4.5:1) and increased further after 4 hours to a maximum Fnr1:Fnr3 ratio of 6:1 (Figure 5B and C). However, an increase in promoter expression was only detectable 2 hours after the switch in the wild-type strain and maximum β-galactosidase activity was observed after 8 hours. In contrast, in the *fnr1* deletion mutant, only partial activation of *pfnr1* was observed (Figure 5A). Since the *fnr1* deletion did not significantly influence the level of Fnr3 (Figure 5C), we can conclude that the *fnr1* promoter is only weakly activated by Fnr3 homodimers. Surprisingly, although transcriptional activation of *pfnr1* is dependent on Fnr3, we observed substantial activation of the *fnr1* promoter, 8 h after the switch to low aeration (Figure 5A). However, the absence of Fnr3 influenced the kinetics of Fnr1 expression, so that Fnr1 was only detectable 4 hours after the switch to low aeration. Nevertheless, relatively high levels of Fnr1 accumulated 8 hours after the switch (Figure 5B and C). Presumably the slow kinetics of *pfnr1* activation in the absence of Fnr3 reflects auto-activation of this promoter by Fnr1. The level of promoter expression in the *fnr3* deletion suggests that Fnr1 homodimers are apparently more competent than Fnr3 homodimers in activating this promoter. Overall these results imply that Fnr1 can play a significant role in auto activation of this promoter, but optimal and rapid activation of p*fnr1* following the switch to low oxygen requires both Fnr1 and Fnr3.

To investigate co-activation further we generated a transcriptional fusion carrying an altered Fnr binding motif (pfnr1*::lacZ) designed to favour binding of Fnr3 to the downstream half-site of the motif and enable binding of Fnr1 to the upstream half-site. The altered sequence lacks the 3′A residue at position 14, characteristic of the Fnr1 binding motif and alters the conserved C or T residue at position 7 in the Fnr3 binding motif to bring the upstream site closer to the Fnr1 consensus (Supplementary Figure S5A). If Fnr3–Fnr1 heterodimers play a significant role in activating the promoter, we anticipated that this sequence change could potentially result in the re-orientation of heterodimers bound to the target sequence. This change resulted in a 30% decrease in transcriptional activation in the wild-type strain but complete loss of activation in the *fnr3* deletion background suggesting that the altered Fnr binding site is not recognised by Fnr1 homodimers (compare Figure 5A with Supplementary Figure S5B). These results imply that the 3′ A residue at position 14 is important for recognition by Fnr1. In contrast, the altered promoter was still activated in the *fnr1* deletion background, suggesting that the altered Fnr binding motif can be recognised by Fnr3 homodimers. However, since the level of promoter activation was higher in the wild-type strain than in the *fnr1* deletion, there remains a possibility that this sequence can also be recognised by reoriented Fnr1–Fnr3 heterodimers.

### Involvement of activation region 3 in transcriptional activation by Fnr

**Figure 6.**
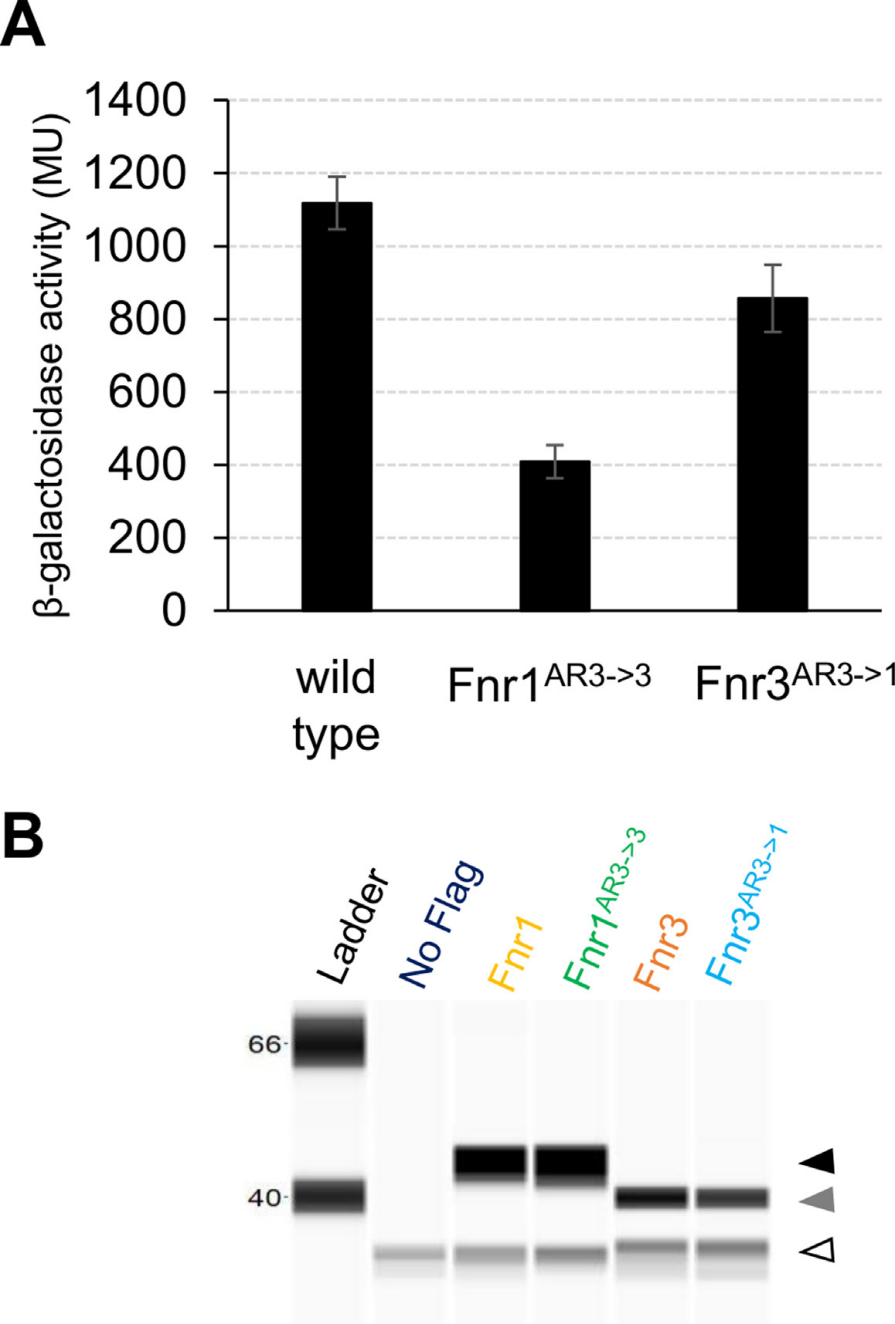
Role of Activating Region 3 in transcriptional activation by Fnr proteins. (**A**) Activity of the native *pfnr1::lacZ* fusion in the wild type strain of *H.seropedicae* in comparison with strains carrying specific activating region domain swaps. The swapped domain strains are Fnr1^AR3→3^ (Fnr1 with Fnr3*-like* activation region 3) and Fnr3^AR3→1^ (Fnr3 with Fnr1*-like* activation region 3). β-galactosidase activity, in Miller Units, was assayed as described in Materials and Methods using cultures incubated for eight hours after the switch from high to low oxygen. The error bars represent the standard error for three independent biological replicates. (**B**) Levels of the swapped domain proteins are similar to the cognate wild type Fnr protein. 3xFlag tagged versions of the Fnr1 (yellow), Fnr1^AR3→1^ (green), Fnr3 (orange), and Fnr3^AR3→1^ (blue) proteins were used to verify the protein levels using the Simple Wes system as described in Materials and Methods. The black arrowhead indicates Fnr1, the grey arrowhead indicates Fnr3, and the white arrowhead represents the cross reacting protein from *H. seropedicae*. The protein extracts were prepared from the same cultures used for the β-Galactosidase activity measurements shown in A.

Fnr-dependent transcriptional activation in *E. coli* is mediated by interactions between RNA polymerase and three activating regions on the surface of the protein designated as AR1, AR2 and AR3 (48–50). All three activating regions are important for activation at Class II promoters where the activator binding site, partially overlaps the –35 region of the promoter (48–51). Determination of the transcript start-site of *fnr1* (Supplementary Figure S6) indicates that the Fnr binding site is centred at -42 and supports the hypothesis that *fnr1* is a Class II promoter (51). Based on the activating region assignments for *E. coli* Fnr, we identified the analogous regions in Fnr1 and Fnr3 from *H. seropedicae* (Supplementary Figure S7). This analysis pinpointed AR3 as the most dissimilar activating region between Fnr1 and Fnr3. AR3 is located within a loop in *E. coli* Fnr and the downstream subunit of the Fnr homodimer contacts the σ^70^ subunit of RNA polymerase to promote transcription activation at Class II promoters (52,53). Sequence differences between the AR3 regions of *H. seropedicae* Fnr1 and Fnr3 may explain differences in their ability to activate promoters and perhaps reflect the involvement of orientated heterodimers if the AR3 region from Fnr1 is more effective in establishing contacts with σ^70^ when positioned in the downstream subunit. Therefore, we anticipated that perturbations in AR3 might affect the activity of promoters in Group III. To test this, we generated Fnr proteins containing discrete amino acid substitutions that interconvert the AR3 regions of Fnr1 and Fnr3 (Supplementary Figure S8). The swapped domain proteins were named Fnr1^AR3→3^ (Fnr1 with Fnr3*-like* AR3) and Fnr3^AR3→1^ (Fnr3 with Fnr1*-like* AR3). To avoid problems that might arise from expression levels, the modified coding sequences were introduced into the *H. seropedicae* chromosome at their respective native locations and 3xFlag epitopes were added to the C-termini of these protein swaps to facilitate immunogenic detection. We compared expression levels of the *pfnr1::lacZ* fusion when activated by wild-type Fnr and the swapped AR3 variants under steady state conditions, 8 h after the switch to low aeration. This revealed that when the AR3 region from Fnr3 was introduced into Fnr1, transcriptional activation was reduced by ∼65% (Figure 6A). This suggests that AR3 of Fnr1 might be crucial for optimal interaction with the σ^70^ subunit of RNA polymerase and hence for full activation of the promoter. In contrast, the introduction of AR3 from Fnr1 into Fnr3 led to a moderate reduction (∼20%) in transcription activation. Hence, although this AR3 swap is not highly detrimental, it does not improve the ability of Fnr3 to activate the promoter. Comparison of protein levels between the wild type and swapped Fnr derivatives revealed no significant differences, indicating that the domain swaps do not influence protein stability (Figure 6B). The results therefore support a model in which AR3 of Fnr1 is required to achieve optimal transcription activation.

## DISCUSSION

Transcriptional regulation in combination with other regulatory mechanisms (54), ensures that appropriate sets of genes are expressed in response to environmental cues, so that bacteria can efficiently use resources to colonize specific niches and outcompete other microorganisms. Mechanisms for transcriptional regulation are diverse and complex (2,55,56) and, in some cases, the occurrence of apparently redundant orthologous transcriptional regulators potentially fulfilling similar functions adds even more to this complexity. In this study, we aimed to understand this potential redundancy by studying transcriptional activation by each Fnr ortholog in *H. seropedicae* using a combination of RNA-Seq, ChIP-Seq and classical genetic approaches.

We found that Fnr1 and Fnr3 regulate specific classes of promoters and may respond to different oxygen levels. Fnr3 seems to be a primary sensor of oxygen status in the cell since it apparently becomes active early after the switch from aerobic conditions to low aeration. Since expression of *fnr3* is constitutive, this activation is likely to involve acquisition of a reduced [4Fe–4S]^2+^cluster. We established that activation of *fnr1* transcription is dependent upon Fnr3 and that Fnr1 is expressed 30 minutes after the switch from high to low aeration. Hence, all genes in the Fnr1 regulon are hierarchically dependent on Fnr3 immediately after a switch to low oxygen. Despite this hierarchy, the regulons of Fnr1 and Fnr3 are discrete in many cases, exemplified by the Group I and Group II promoters, that have distinct DNA binding targets and specific Fnr dependencies. We anticipate that Group II promoters activated by Fnr3, reflect the need to reconfigure metabolism under intermediate oxygen levels, when for example, expression of the *bd-*type quinol oxidase, encoded by the *cydAB* genes is activated by Fnr3 to provide a respiratory oxidase to support growth. Although activation of Fnr3 will trigger expression of *fnr1*, it seems likely that Fnr1 itself will only be activated under low oxygen conditions, reflecting the need to express Fnr1 targets, such as the *cbb_3_* high affinity oxidase, encoded by the *fixNOP* operon. Therefore, the regulatory hierarchy appears to involve Fnr3 as a global sensor of the adaptation to low oxygen levels, since it is required to switch on the expression of not only Group II promoters, but also the promoter of Fnr1, which will then expand the regulatory cascade to promoters in Group I. Any differences in oxygen sensitivity between Fnr1 and Fnr3 are likely to reflect differences in residues flanking the conserved cysteine residues that ligate the [4Fe–4S]^2+^ cluster, with consequent effects on cluster stabilisation (20,47,57).

We also identified a family of promoters (Group III) such as *fnr1* that require co-activation by both Fnr1 and Fnr3 for optimal expression, indicative of more elaborate fine-tuning. Although these promoters can be activated to a certain extent by a single Fnr protein, full expression was only observed when both transcription factors were present. The transcriptional activation mechanism at these promoters may involve heterodimer formation, since we detected interaction between Fnr1 and Fnr3 *in vivo* using the two-hybrid system in *E. coli*, and this interaction was modulated by substitutions in the dimerization interface. We only observed dimerization under anaerobic conditions, reflecting the requirement for the reduced form of the [4Fe–4S]^2+^ cluster to enable formation of the coiled coil interface in the dimer (43,45–47).

Group III promoters such as *fnr1*, may enable low level expression activated by Fnr3 and subsequent higher levels of expression, when Fnr1 is activated, presumably in response to appropriate oxygen levels. We envisage two different models for the mechanism of co-activation of promoters by Fnr1 and Fnr3, which are not necessarily mutually exclusive. In the first scenario, initial binding of Fnr3 homodimers ‘pump primes’ transcription, prior to the activation of Fnr1 at lower oxygen tensions, when Fnr3 is subsequently exchanged by Fnr1 homodimers at the Fnr site resulting in a higher level of promoter activation. This exchange could be facilitated by the 5-fold higher level of Fnr1 compared to Fnr3 under these conditions. In the alternative model, Group III promoters are activated by orientated Fnr3–Fnr1 heterodimers, in which Fnr1 is located in the downstream subunit, favouring recognition of the Fnr target sequence and hence improving recruitment of RNA polymerase. In both models, it is anticipated that optimal activation of the promoter is achieved, when AR3 located in the downstream subunit of Fnr1 contacts the σ^70^ subunit of RNA polymerase, in line with previous observations for Fnr and CAP proteins in *E. coli* (51–53,58,59). This is supported by the observation that substituting the AR3 region of Fnr1 with that of Fnr3, decreases the level of transcriptional activation, similar to that observed with the Fnr3 homodimer. Both models provide a means to integrate activation of Fnr3 with that of Fnr1, providing increased levels of expression according to physiological demands. Thus, autoactivation of the *fnr1* promoter provides a burst of Fnr1 expression that favours transcription of discrete groups of genes belonging to the Group I and Group III categories, including those that are essential for efficient adaptation to limiting oxygen conditions. This suggests a fine-tuning mechanism for gene expression in which electron flux is optimised towards particular respiratory oxidases in response to fluctuating oxygen concentrations.

## DATA AVAILABILITY

The RNA-Seq and ChIP-Seq data are available at ArrayExpress under the accession numbers E-MTAB-5741 and E-MTAB-6395, respectively.

## Supplementary Material

Supplementary Data
