# Supplementary material for: Hierarchical interactions between Fnr orthologs allows fine-tuning of transcription in response to oxygen in Herbaspirillum seropedicae
Source: Nucleic Acids Res. 2018 Feb 26;46(8):3953–66. doi: 10.1093/nar/gky142 (PMC5934665; doi:10.1093/nar/gky142)
Supplement: Supplementary Data [file gky142_supp.zip › nar-00262-v-2018-File008.pdf]

# Supplementary Material for:

# Hierarchical interactions between Fnr orthologs allows fine-tuning of transcription in response to oxygen in *Herbaspirillum seropedicae*

Marcelo Bueno Batista[1], Govind Chandra[1], Rose Adele Monteiro[2], Emanuel Maltempi de Souza[2], Ray Dixon[1*].

[1] Department of Molecular Microbiology, John Innes Centre, Colney Lane, Norwich NR4 7UH, UK

[2] Department of Biochemistry and Molecular Biology, Universidade Federal do Parana, P.O. Box 19046, Curitiba, PR 81531-990, Brazil

Supplementary Figures S1 to S8

Supplementary Files S1 and S2

Supplementary Tables S1 and S2

Supplementary References

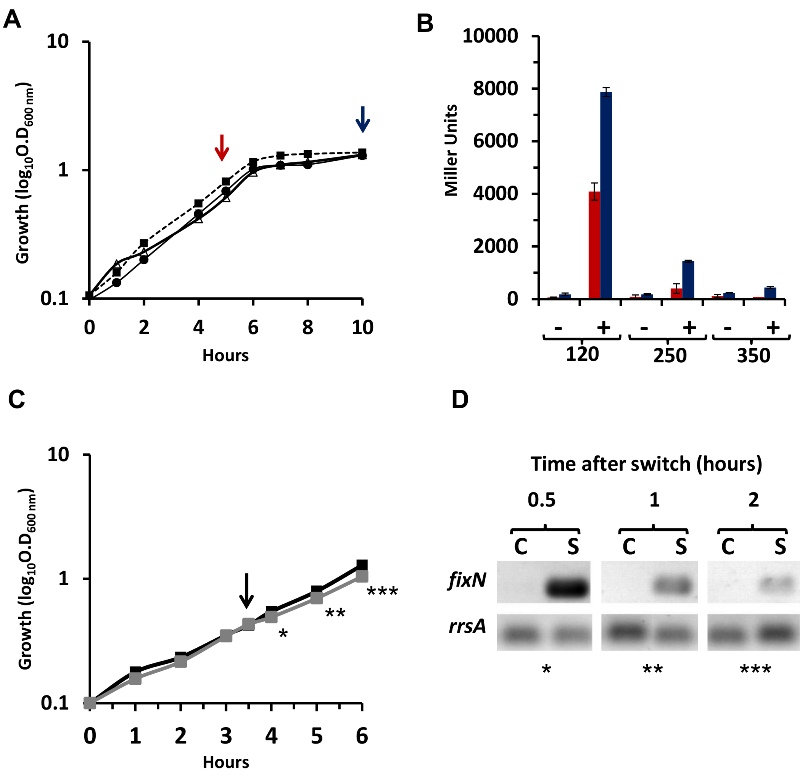


**Figure S1. Fnr1 is activated 30 minutes after the switch from 350 to 120 rpm.** (A) Comparison of the growth profile of the *H. seropedicae* SmR1 strain under different aeration regimes. The growth curve was performed at 350 rpm (squares), 250 rpm (open triangles) or 120 rpm (circles). Arrows indicate the time points when β-Galactosidase activity was measured. (B) β-Galactosidase activity of the *pfixN::lacZ* fusion (+) under different aeration regimes as indicated. The red bars indicate activity in the exponential phase of growth (red arrow in A) while the blue bars indicate the activity in the late stationary phase (blue arrow in A). The promoter less vector (-) was as used as a control. (C) Growth profile of the *H. seropedicae* SmR1 strain at 350 rpm (black line) and after the switch from 350 to 120 rpm (grey line). The arrow indicates the time of the switch and the asterisks the points of sample collection for RNA extraction prior to performing RT-PCR. (D) RT-PCR of an Fnr1 target gene (*fixN*) after different times after the switch as indicated by the asterisks in (C). Samples were collected from cultures grown at 350 rpm (Control-C) or after the switch from 350 to 120 rpm (Switch-S). The *rrsA* gene, coding for 16S rRNA was used as an endogenous control.

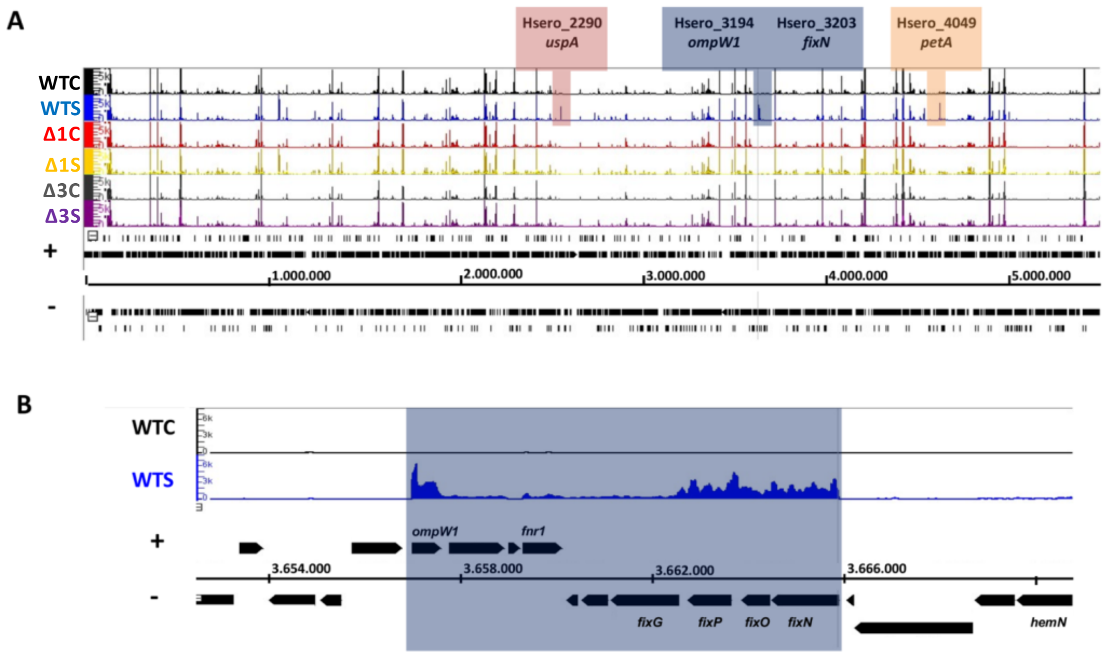


**Figure S2.** Overview of RNA-Seq data used in this study. (A) Genome-wide view of transcripts mapping to the *H. seropedicae* genome under different growth conditions are shown using the IGB viewer (1). The lanes are colour coded as follows: WTC, wild type under 350 rpm (black); WTS, wild type after switch to 120 rpm (blue); Δ1C, *fnr1* deletion under 350 rpm (red); Δ1S, *fnr1* deletion after switch to 120 rpm (yellow); Δ3C, *fnr3* deletion under 350 rpm (grey); Δ3S, *fnr3* deletion after switch to 120 rpm (purple). Groups of genes that are highly differentially expressed after the switch to low oxygen conditions are shaded in different colors for WTC and WTS lanes. (B) Zoomed in view of the most highly induced genes (shaded in blue) in the genomic neighborhood of the *fnr1* gene.

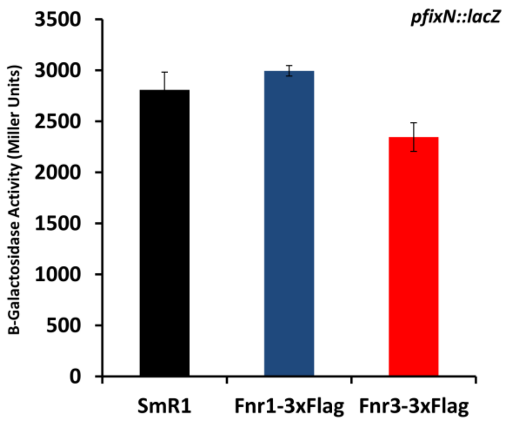


**Figure S3. The activity of the Fnr1-3xFlag and Fnr3-3xFlag proteins is similar to the activity of the native proteins.** The *pfixN::lacZ* fusion was assayed for β-galactosidase activity in NFbHP-Malate media supplemented with 20 mM of ammonium chloride 4 hours after switch to low oxygen in the *H. seropedicae* wild type (black bar), Fnr1$^{3xFlag}$ (blue bar), and Fnr3$^{3xFlag}$ (red bar) strains. Note that activation of the *fixN* promoter requires both Fnr1 and Fnr3. The error bars represent the standard deviations from two biological replicates.

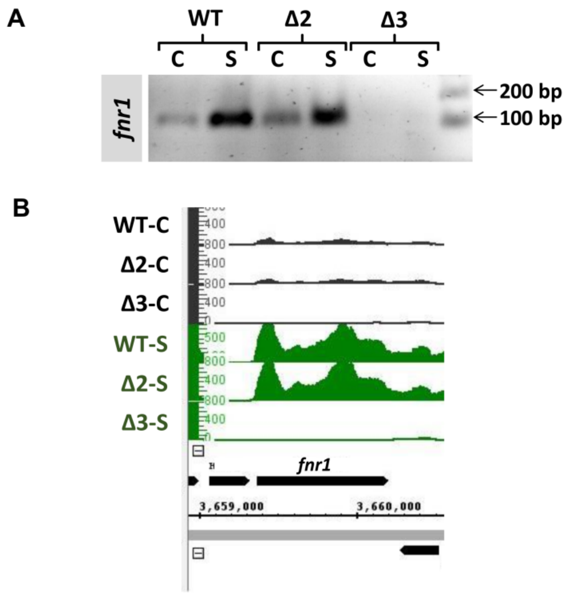


**Figure S4. Expression of *fnr1* 30 minutes after the aeration switch is dependent upon Fnr3.** (A) RT-PCR targeting the *fnr1* gene was performed using RNA samples from different strains of *H. seropedicae* SmR1 (WT), *Δfnr2* (Δ2) and *Δfnr3* (Δ3) prepared using cultures harvested 30 minutes after the switch form high (C) to low (S) oxygen. (B) Genomic view of transcripts mapping to the *H. seropedicae fnr1* gene in different strains are shown using the IGB viewer (1).

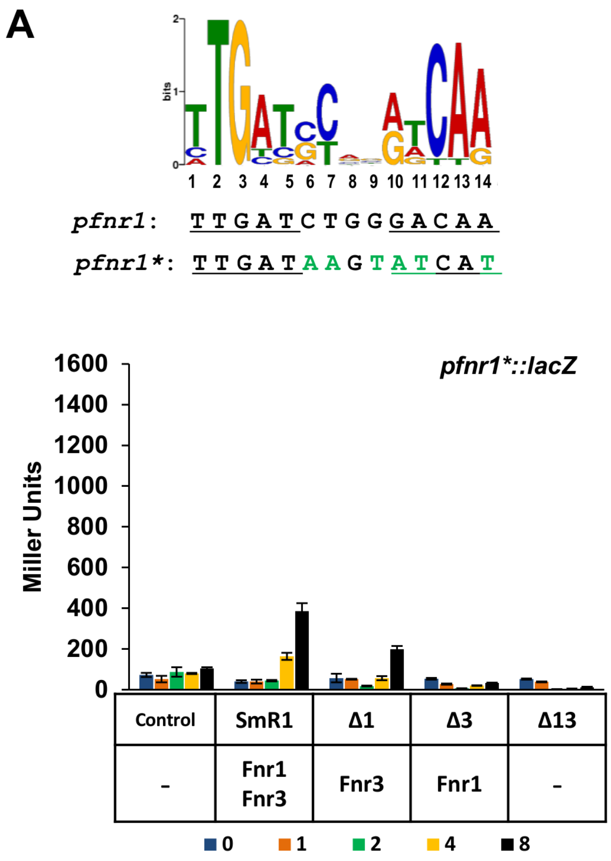


**Figure S5. Influence of the Fnr binding site sequence on promoter activation.** (A) DNA-binding motif of Group III activated promoters with the sequences of the Fnr-binding sites at the wild-type *pfnr1* promoter and the modified Fnr binding site at the *pfnr1** promoter shown below. Base changes in the modified Fnr binding site are shown in green. (B) Activity of the mutant *pfnr1*::lacZ* fusion, carrying the altered DNA binding motif, in different *H .seropedicae* strains. The *H. seropedicae* strain used, as well as the Fnr proteins expressed in these strains are indicated below the graph. Control, denotes the activity of a promoter less *lacZ* plasmid (pPW452) in the wild type strain of *H. seropedicae,* while the minus symbol ( - ) indicates no transcriptional activation. β-galactosidase activity was assayed as described in Materials and Methods using cultures incubated for zero (blue), 1 (brown), 2 (green), 4 (yellow), and 8 (black) hours, respectively after the switch from high to low oxygen. The standard error is representative of three independent biological replicates.

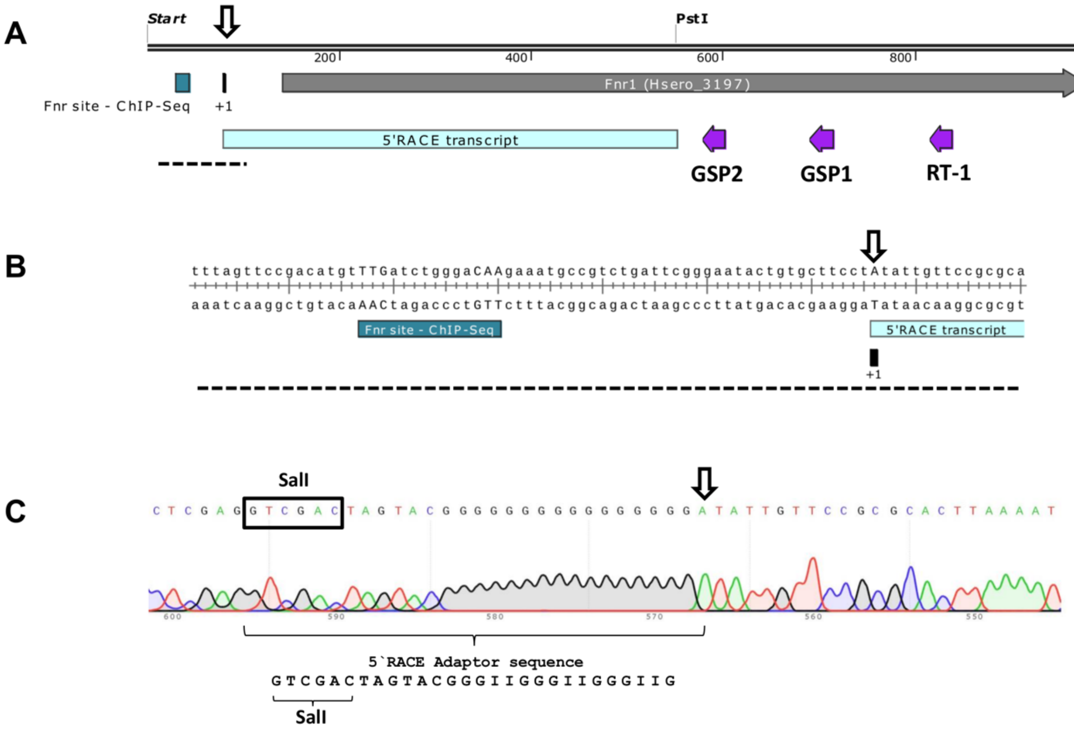


**Figure S6. Determination of *fnr1* promoter transcription start site.** The experiment was performed using the 5' RACE System for Rapid Amplification of cDNA Ends - version 2.0 (Invitrogen #18374-058) as recommended by the manufacturer. (A) Map of the *fnr1* gene coding region and its promoter. The Fnr-binding site predicted from the ChIP-Seq data and *fnr1* coding sequence are depicted as a blue box and grey arrow, respectively. The 5`RACE transcript (light blue box) is aligned to the sequence and the predicted transcription start site (+1) is indicated by a thick open arrow. The primers used for the RT-PCR (RT-1), gene specific PCR (GSP1) and nested PCR (GSP2) are represented as purple arrows and its sequences are shown in Table S2. The dotted line highlights the zoomed in region shown in (B), which spans the beginning of the 5`RACE transcript alignment and the *fnr1* promoter. (C) Sequencing electrophoretogram for the 5`RACE transcript. The nested-PCR product was cloned as a SalI/PstI fragment into the vector pBlueScript KS II +, linearized with the same enzymes. The PstI site, from the *fnr1* gene, and SalI site from the 5` RACE adaptor are highlighted in (A) and (C), respectively.

```
Fnr-Ec       ------MIP---EKRIIRRIQ------------SGGCAIHCQDCSISQLC 29
Fnr1-Hs      MNQSCHSTPITTPANAAAPLTHCSSLTAAREAAASSALRSCTACGMHQLC 50
Fnr3-Hs      ----MSSLPASTHKHSPPPVN------------LHALRASCSACSMHQLC 34
                     *     .    :               .    *  *.: ***

Fnr-Ec       IPFTLNEHELDQLDNIIERKKPIQKGQTLFKAGDELKSLYAIRSGTIKSY 79
Fnr1-Hs      LPMGLDESDMKRLDKIIGRRK-VARDDFLYRIGDRFTALYAVRVGHFKTY 99
Fnr3-Hs      LPMGLDQGDMQRLEQVINRRRKVKRDETLYRLNDKFDMLYAIRLGHFKTF 84
             :*: *:: ::.:*:::* *:: : :.: *:: .*.:  ***:* * :*::

Fnr-Ec       TITEQGDEQITGFHLAGDLVGFDAIGSGHHPSFAQALETSMVCEIPFETL 129
Fnr1-Hs      QENLDGDRQITGFQMPGELLGMDAISTEQHQCDAVALQDSEVCEIPFARL 149
Fnr3-Hs      QHNPNGGQQITGFQMAGELLGMDAIGAGHHLCEAVALEDSEVCEIPFASL 134
               . :*..*****::.*:*:*:***.: :*  . * **: * ******  *

Fnr-Ec       DDLSGKMPNLRQQMMRLMSGEIKGDQDMILLLSKKNAEERLAAFIYNLSR 179
Fnr1-Hs      EQLFGQIPHLLRHFHRIMSHEITSEQNVIMLLGNMRAEQRFAAFLVNLSS 199
Fnr3-Hs      EDLFRDMPTLLRQFHRMMSLEISREQRVMLTLGSMTAQQKMAAFLLNLSS 184
             ::*  .:* * ::: *:** **. :* ::: *..  *::::***: ***

Fnr-Ec       RFAQRGFSPREFRLTMTRGDIGNYLGLTVETISRLLGRFQKSGMLAVKGK 229
Fnr1-Hs      RYAARGYSSTRFQLRMTRQDVGNYLGLTIESISRLISKFRKQGLLAVEQR 249
Fnr3-Hs      RYMSRGYSSTRFQLRMTREEIGNYLGLAVESVSRLLTNFKKSGVIEVNHR 234
             *:  **:*. .*:* *** ::******::*::***: .*:*.*:: *: :

Fnr-Ec       YITIENNDALAQLAGHTRNVA-------- 250
Fnr1-Hs      DVEVVDLAALKRLAAGVDACTATPTRSTS 278
Fnr3-Hs      DVELCDLPTLRAVALGNDPCA-------- 255
              : : :  :*  :*       :
```

**Figure S7. Alignment of *H. seropedicae* Fnr1 and Fnr3 proteins with *E. coli Fnr*.** Identical amino acids are indicated by asterisks ( * ), high similarity amino acids are indicated by colons ( : ) and low similarity amino acids by dots ( . ). Conserved cysteines required for binding of the [4Fe-4S]$^{2+}$ cluster are colored red and highlighted in yellow. The dimerization helix is in light blue and the helix-turn-helix domain is highlighted in grey. The AR1 adjacent loops, residues 71–75, 116–121, and 184–192 are shown in green. The AR2 residues 49-50 are in dark blue and wave underlined. The AR3 residues 80 – 89 are shown in red. All numberings are relative to the *E. coli* Fnr protein. Targeted amino acids in AR3 (red) and the dimerization helix (light blue) are indicated in bold and underlined font.

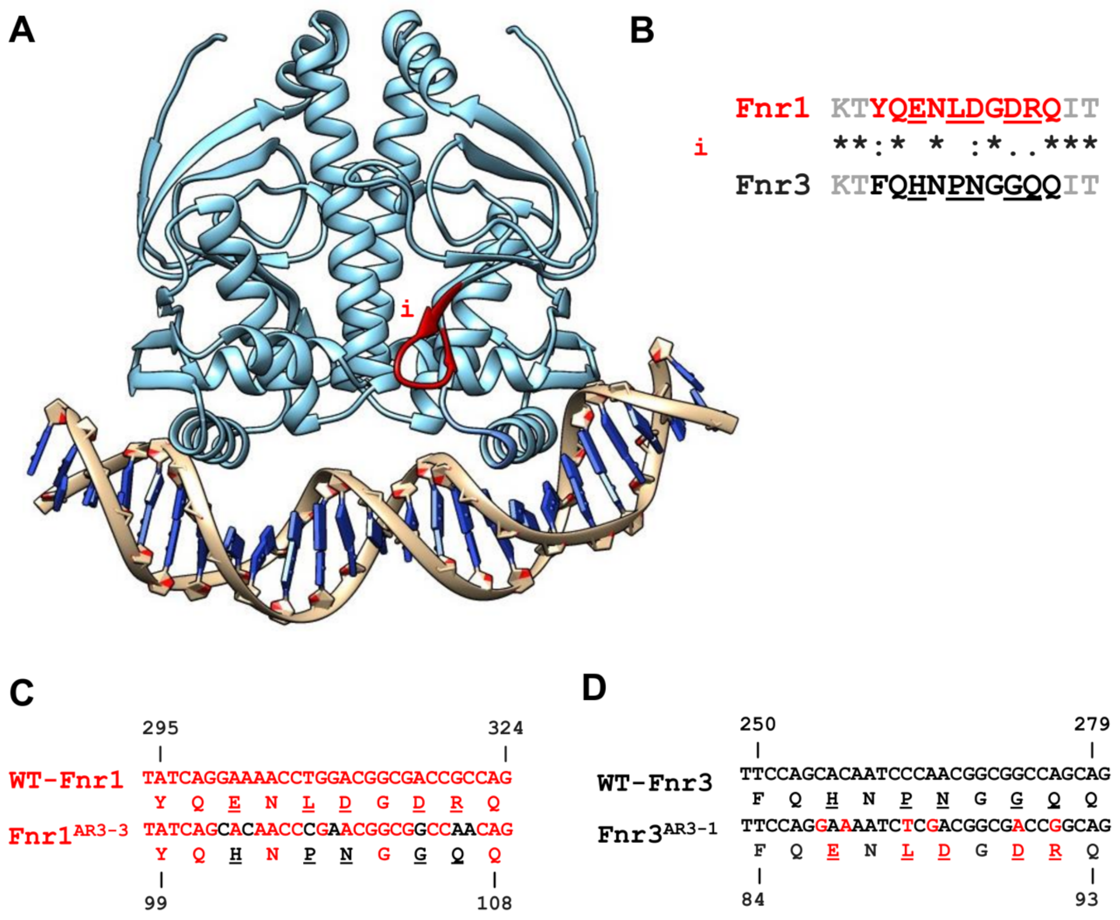


**Figure S8. Structural visualization and comparison of activating region 3 (AR3) in the Fnr proteins from *H. seropedicae*.** **(A)** Dimeric structural model of *H. seropedicae* Fnr1 generated using Swiss-Model software (2) with the crystal structure of *Aliivibrio fischeri* Fnr (PDB: 5E44 (3)) as template. AR3 is highlighted in red and indicated by the letter i. (B), the AR3 amino acid regions (i) of Fnr1 (red) and Fnr3 (black) are aligned with residues swapped between the proteins as indicated by the underlined amino acids. (C) and (D), DNA and protein sequences of the AR3 regions in Fnr1 and Fnr3 respectively, with changes to generate the protein swaps indicated by color coding. A full alignment of *H. seropedicae* Fnr1 and Fnr 3 is presented in Figure S7.

**Supplementary File S1**

List of sequences used for MEME search

**Group I**

```
>Hsero_0239
tacaagcgcgcagcctatggtgcggccttcctcctctatcggacggtaattcacatcatc
acgctgggaaaagatgcctgagatatatttggcgtccgcttgatttaccgaaaattcacc
gtatcgcattccccgctgaaatggaataagatgaccgctccggtttccggcggggagccc
agcggcttcccgtcaatttctaataaaggagcgagttatggatgatgtggtcattgtcg
cagcacagagaacggcgatcggcaaattcggcggagccttgtcgaagattgcggcagccg
a
>Hsero_0260
agaattctcatcaaaagccgggcgcgattgcgatgcatcaacgcggtattggcaggaaaa
cgacaagcttgcctctccgcaaccctgcaaggagccctacgatgaacccggacaatggaa
actggctatcggccgaacaagcggtgttgcccgacggcacgctgcgcgagaacctgccgc
tggtgaagaagaagctgcccctgttctcgccccagcaggtgcaagcagagccgcaggggc
agcccgacaatgaggagaatgtggcgccgtcgccgcgccaggactgagagccacgccaaa
a
>Hsero_0262
ttttggcgtggctctcagtcctggcgcggcgacggcgccacattctcctcattgtcgggc
tgcccctgcggctctgcttgcacctgctggggcgagaacaggggcagcttcttcttcacc
agcggcaggttctcgcgcagcgtgccgtcgggcaacaccgcttgttcggccgatagccag
tttccattgtccgggttcatcgtagggctccttgcagggttgcggagaggcaagcttgtc
gttttcctgccaataccgcgttgatgcatcgcaatcgcgcccggcttttgatgagaattc
t
>Hsero_0371
tttgtttccagatgtgtccgcacgtctgcagcggccctgccggatcggcaggtttcctct
tgtctgacgcccattggcgagcttctctgatgccgcgcaaggcgacgcctggttatcctg
cttattctgatcattctgcatgatgtgtcggcgctgcccgtgtaccgatgtaccgacact
cacctccccacccgtagaactgaagtaaacagaactgaacaagcatcagcaggtctatta
ccatgccatccaatgatgagcttgcgcgctcattgccaagagaaaggaaatgacatgccc
a
>Hsero_0624
tgggcctggccgagaaggccggcggcctgttgaaccagatggtcccgtcgatccgcaaga
ccgctgacctggtgcaggagatcgctgccgcctcgcacgaacaatcgaccggcttggagc
agatcaacaatgccgtgcaccagatggcgcagactacccagatgacggcctcggcctcgg
aagagctgtcggccacttcggaagagatgagcgcccaggccatccatctgcaagacctga
tgcgtttcttccaggtcggcgagccgcgcaagaacgccccgcgcgccagcaaggcggtgc
c
>Hsero_0660
aagcgccgccggcagcaactggcgggctagcccgcccttgctgccgagggcggtggccg
aagtttttcccgaagtattttcaagtcttgcgcggcgttgacccaggtcagcgccaacag
gcatcaaagcgctaagctttgcaacttattcgctagtgcgtatttccggccttgcccata
ctggcaacgccctttcattttcacgaggaacccatgaaagcagccgtcacccgttcatcc
caacaaggcttcaccctgctcgaactgatggtgacgctggccatcgtcggcatcctggcc
a
>Hsero_0835
gctgaggcgtcggccggatcggtccgtatcgacccgtatcggcccgtatcggcggaatgc
aagaaatgcactttcccggtggaaaaacaacagcacagttgaatgtgagcgcaaacattc
caagtattttgatgcagcgcaataagctgacgatatactgacgcattccccagcagatac
cgcgatttgacatacgcgcccggagcaatccagggcgcagggctagtgcgttcttcgctt
tcgttgcctcctggacaacaaaggaaaacagatgggattcaagctcgccgatcggggcga
g
```

>Hsero_1002
gccgctatcggcgcgttgtcatggccaggctcaacagcggcgcatccagtgcgagtgcgc
cgccggcttgccgcgccagccctttctggtggggccatgcagcggatgagtgaagcttaa
gagtgagcttgatattgatcaaatggattgttttgattcatgatatggacggcatcaata
cattgaccaccccatccgtcgtctatccgagggtcggcaagaacaagcacaacaacggag
accagcgccatgcgtttcaacaagctcgacctcaatcttctggtcgccctggatgcactg
c
>Hsero_1165
gaacgtctgcgccgcgctggcatctacggctttgcccgctgctggccgggcgcttgcgca
acaggaagcattcgcattgccgcccgccatacgggcgcctgcccggcgaagacggcattt
gatcgagtgcaaagaattgtcttccgctttagaatctcaggttctgaatagaaccttcgt
agcaaaccaacaagactgaaaggattcatcatggcacatacccttccggcactgccttac
gatctggacgcgctggcgcccacgatttccaaggaaaccctggaatatcactatggcaag
c
>Hsero_1250
gtttccccgttgggcgggacgcgcccatggcatcatccgcatccgtcgccactgccgccg
ggtcggtggcgaagtaatggaatggattttgctaaacctcaaaagcagcactgatcacag
cacctatagttgtgaatgcgcgccgggggggggcgttgccatcaacgtcggcagagacag
taacaaccataaggggggaatcatcatggggcaattgttggccaatctgaaactgtggcaa
aagtttgccttgctgggactattgggcctggtgttgttcggcgtgccgacgaccttgtac
t
>Hsero_1337
caggttaaggcatgcccatggtgcagacaatgcgtgccacatgcccgccccaccggggat
tgcggcagaccatctaggtaaaaatacctagatcacacccccggtggcagtgttaaagtg
tcgctgatcccaagaatttcaggcctgtttttttgcatagatcaaaacgtggtgttttgat
ttgtgccgaccggaccatcaagaaatcgagagggtcggaaatagcgccggtgcacaccgg
ctccattttgacgagacgaggagtaatgatggaaaccaacccgccggtggtctacatcgt
t
>Hsero_1446
gctggaaccgtgcgcgccaggtcgtgacccaggcagcctgatcactctcctgccgcttca
catcatggacggaccgggatttcccggtccgttttcttttgggcgcttgatatgggtcag
agcaggatgaatatgtatcttcattcatatagaagccatctggcggcttacaaatgctca
catccccttggtgtagaatccccgcttgctctgcgcgaacgccggtacggccggttcccg
cagcccccgggaataggactcaacagcctgcatcttgcgccttgcggccgcctttgccgt
t
>Hsero_1880
Gctcggccctgggcggttgggccggactgcgccatgtgctgcgccgtccacccttgcaga
Ccctgcgcgagggctgatacgcgccaggtcaaatcgcctgcatatcgccgccgggcggcc
Cgccgtgggctggccggctgcggtatcatgacgctcttatgcaaatcgaagaccccaacc
Aacccaccaccttcgaactcatcggcggcgcggagaaactgcgcgagatggtcgatcgtt
Tctatgacctgatggacctggagccggagttcgccggcatccgcgccctgcatccgccct
c
>Hsero_1552
ggcatagaccgtggccaccgagcggctcaccgcttcggccagctgcgccagctggtaggg
aaagaagtcctgtagtttcatgcggacatggtaacagacgttaccaaaacacgacatcca
gtgccggcttgatgcacatcaagaacgataactttgaagcgcttcatctcgtcatcgacg
cattgaccatgccgtggctcactccatatagtaacaaatgttactaattaggccgcatcc
ggccatggagaccaatgatgaacatcgaacgcatccaccacgtggcctaccgctgcaagg
a
>Hsero_1957
cctgagcgtgctgctcatcacctcagggctgcaaggtggggatgtcggacgagcaaaaga
gaacgttcttgcgacaaaaattcatgaaaatcaaaatgatgtgattgactgattgccacg
tagcactttccctactacatttgtgaatcaggcatttgcctgtcacatcatatccaacgg
ggagtgggggaaatggataatttccaaaaagagattgatgaaagaaccaatctcacggca
tctaacaaattcgagctgttgctgttccggttggggaccgatccgcacggcgagcgctct
g

>Hsero_1976
aaggaagcgcactggcggcttcctttttgttttgtgcctctctttttctctccccgcac
tttcctgctgtgcgtgacctgccgtgatctgtcatgggccgggttgtcctcgatcaatgc
ttgcgcaatgacagccgggtaaaacctcgtttttgcgctggtccggcctgatgtttcacg
gaagcatcgaaaaatgaattaaacttgctaacgaatttttttgcgagttttgtcctcgaac
tcctctctcaccgaagaaaccaaattagctatggcggcagatgatcagtatttcctggta
g
>Hsero_2144
agttggcgcacaggcaggcaacgacccaagtgtcgttgcgaagcgcctcggcgagttggc
ggcggttggtctggtctaggatctggtaagacatggcccgcgtatttaaccgcttcggc
agcagccggtttgcgctggcgcacactacgtcatccttcacgcaggcaagccgaagaatg
ctgcgcgcacggtaaaatagcgggatgaccacgattcttgccattgaaacttcgaccgag
ctcgcctctgcagccttgctgtaccagggcaagctgctgtcgcggcagtcggctggcgcc
c
>Hsero_2379
tgatcctgttcgggtggggacgcgcgatggatggaaaactcatttcgtgagaccaggtct
tgttcggcagtgtcagggcatcggatatcgaccacgcacccggacattgctcggagattt
ttgatacccatcaaagattcacattttgcgctgcaccaggtatcgctatgatgaacaaag
caagatggagccggaaccctacgaacccggaccagagcaggccgggggctatggcgctg
gggaaacctagctcttgctgcaagcaacggaggaccttgcaaatgcattctcgtcaatca
a
>Hsero_2384
tgttgcacaaggctgagcaagcagggcaacgtcgtccccgacaggaccggtctgctggtt
ggaacatctgcccgcttggccgcagccttttcgctggattcgcccaattccctttgacct
ggatcagagggaagggcccgcaggacatctaatctggaaagatattgctcttcgttgcgt
cccctatccatttccctcgcgagaggcttgcctatgagtgtcagcaatcgactttatcag
gccatcctcgtcttctgcgccgtcaccttggcagtgggcatcctgggccgatggatgggc
c
>Hsero_2403
gcgaggtcctgggcctggatgcgctggcgtagcgcggactctgccgtatccagcagagag
Cgcagccgtaccagctgttgtgtcgaaaggtgtcgcatgaaagctccttgtctggccatg
Cgcgccggggagtcgcgtcatggcatggacactttgcgccatgcacggtatcgcgccact
Gatttaggtcaaagtgcgcagtgctcaccctctggatcgacggccctgaatttgacctgc
Atcagttttgccagcggggcccttcgtagaatcctttatatacggtgcagaagtggtcag
c
>Hsero_2404
gctgaccacttctgcaccgtatataaaggattctacgaagggccccgctggcaaaactga
tgcaggtcaaattcagggccgtcgatccagagggtgagcactgcgcactttgacctaaat
cagtggcgcgataccgtgcatggcgcaaagtgtccatgccatgacgcgactccccggcgc
gcatggccagacaaggagctttcatgcgacacctttcgacacaacagctggtacggctgc
gctctctgctggatacggcagagtccgcgctacgccagcgcatccaggcccaggacctcg
c
>Hsero_2458
gcgggcgcacgcgtgacgctggagctgccgctgtcggccatcagcctggaagccgacgcc
gagcctgtcgtcggcagcgcgacacccgcttttccgagcggtgcttgatgtgccgcaagc
caggcctgaccagcctttgacgcgaatcatttcctttttcttctgatggcggcaatgccc
ggggccgtctcgccttacaatagcgccttgggaaagtagaagaaatagggaatgcaacat
gccggctgatcgcctgcttctgatcattgaggatgacgccgccttcgcacgcaccttggg
g
>Hsero_2909
aggtgcatgtgtaggcggcaaacctgttgcacagctactgcgcgttgagatgacaagctg
cgtgacggagcgagaaaattcccaattgatcaaagaaaagatttcttctatttgatatgt
agtatccaagtgcatttgattttatgttaattttcgatggggtagtacatgcttatacgt
ttaatccatttctcataggttcgtatcgcttggtcgaatagcgaactgagtaacttaagt
tttggcgtgggcagggaggataattttgtttgcttgaggggggggcggcatgacgagtcta
a

>Hsero_2999
tgtctcggtatcttgtccgccgtcctggtgctcgcgtttcatcgtgctgtcatatccggc
ctcttccttgcggccagctttcaatttcgctttcttttgacgattccgcttgcgttgcgg
cgtacccatcaggtataaaacaacggtcaagggaaatatcccgtacagcaggaaagtggc
tacgccagcgactgctgactgttcggtgatcgacatcatgaagacgacgtagagccacgc
gatagcgacgatgtacatggtgatatggcgcttcgatacaaaccgtaaagatacgcgaaa
a
>Hsero_3072
gctcagcatgatgagcagtcagcgcagcgccgccacccctcccaccttcattcgcatatt
cccgtgatgaagcaagaggaagcgaactcccgtactacctcactacattcccccatggca
agattgccgaaatgaattttgctcacgtattgacatatcgcaagcatcgccggctggcct
tccctgcattcgcgttataaagctagcaattctcgttgcacaacgattgcagcgagaaaa
tcctcgtccgaaaagagacggcacaccagtagctggaggaatgaatgatcgtagtacaca
t
>Hsero_3111
ccgccaggccatcccggccaaccggcaggcccgcgccgacgccctgacccgcctgggtcc
gtgggccgaccaggtgccgccggccacgctgccttgatccgccgcaagcgcagcgcgtgc
gccttccggcaggcggaataattctcgactaaactcctaggtcttttccggaacaccgac
gcctgagggcggtcggtatgaaggaacatgcattgcagcaaccgtcattccaacagccgg
gcgcgccgccacgggaagcccagcgcagcccggctccattcatccgccaacgcgacaaga
a
>Hsero_3194
ctgccggcctttacagcacaacggcccggacctgtcatcgcaggtccgggccgttcagct
tttgcctcatcggggtgcagccatggccgcgcttgatcggcatcaaagccggacagccac
ttcacgcatacctttcgcacatcctcactcgacaggtaaaaaccgtgaaaaaaatcgtca
ctattgctgccgctgctgcggctttcgcttcctccttcgctgcccccgtcttcgcccagg
aggcccagagcccatggctggtgcgcgtgcgcgccgtccacctgagcccggaaaacaagt
c
>Hsero_3203
cgccgtcccgcaagagccatccggcggcgcgtgattgtgtgcactgcggttgacgcagac
cgttgtttccccgtctttgttcccgcgcttctccccattttgatacacgtcaaggctgcc
ggaagctgcgccccgtaagctcgcattgaccagtactcaacatagtggggagagtttcgt
gagcaaagaaaatagctacaactacacggttgtgcgccaattcaccgtggcgaccatcct
gtggggcgtagtcggtatgttggtcggcgtgatcatcgccgcccaactggcatggcctga
a
>Hsero_3207
atgcagggccggttcagatcagaggctaaggagttgcctgggggaagtaaagcgcgacag
gtgcaaaaaagcggcgcaatgctgcattcctgccacgtcaggccttttgatacacatcaa
ggtgtttttgcgggggagccgatatcctgcaagctccagtcaataccagctcgcccggtg
ggctcgcggcccagcgcatgtgcgcgccgcgtcagtccggcgtggcagccggccaggagc
atcgcatgcaagtcgcccatccgtccaccctgcagtcttccgccagtctcgccggcatga
a
>Hsero_3246
attggagtccaatcactactcctctctgttgtatttccgggctgcggcaatatgtcgcag
ccctttttttatcctgcttgtcggtgctaaatccttgtggcgcaaggagattttctccgg
gtaggcgtttgcctgccagatggcggcatggcttccgcattcgcagtgatgcgcctgcat
gctagattgcgcggacaaaaaacggggagtgcgacgcctacggcagtcgcgggtgcgatt
gcgcgcattgcccgacaagatcaggtttccttcgccatgaacccattcccccctgcgcagg
c
>Hsero_3273
cgatagtggtgcgggagcggcaggcgctgcggtctggtcgcgcggtacgcctgccacccg
ggttggggcctaagaaccatccccctgccattcccgcaaggcaaggactctttcaaaacg
gtttctcgactccgtgattatacctgcatcaaacttggtggctgccaatggcttgctgct
gtgccagcccgcctataatggcgggcatgaaaaaactcgatctgctcggcggcatcacgc
ccgcccaattcctgcgtgactactggcacaagaagccgctcctgatccgcaacgccattc
c

>Hsero_3488
ttttgtttaattactcgtttttattgtggaataacacacacacttttgctcttagggaaa
attcgagaactccatatggtctgatcttgaagggtttagccaggaaggataacactcgga
tttttgatatggattaaatagctcgcattcaatttcaaccataaaaggggggttccatgtt
caaacacatgaaaatcggcacgaagcttgcgctggggttcggggcgctggtcgttctcac
cattctcgtctcgggcatatcgttaagcagtattcgcgccctgacaagttcgatcaaagc
c
>Hsero_3532
attcgggcggtcggatgttttgtcgtgtgagcaggaatgcaaggattttgtgcggagagt
gtggaggacgtgtggaggtgtcagtgttttgtcagtcattctgcctgcctgtcagcgttc
gcgacgcagcgaaaatcccccataaaattgcgagtcagttttgcttgatgtcaagatttc
tgcacattgcccagcgaccgcaaaggaatgctaaggcattcttgatgctggcaaaactct
gttcgcgtgcgcaggttttcatgtcggacctcctagaatcaagctgccattcgggcatag
a
>Hsero_3853
cgcgcacgcgacttcctggccaagatcatcacgcactgatcgcgccagcctgtcctgcag
caagggccgctccggcaacggggcggcccttgtcgtttgggctatatcaaaagcgggcag
ggcgggcgcgccggggtgcttgacggctacggtagaatgtcgatcctgaagacatcgcgg
ctggctctccacagcccgcatccgccgggatgcctcgcctgatccgtcatcctcggaccg
acacccacaccaagaccgaaagatccgcaatgagcaacgtactgaccatcacccgcccgg
a
>Hsero_3886
ccttcttatcgatgaatcgggtggcagtgacggcgtggcagcaatccgtcatggcgaacc
gcaagaataaggcaaaaatcgggaggaagtccgacctggcgcaatgactgtccataattt
catgcaggcccggcggtgcaagactatatttgagtcatcccatcagcgcccctgggggcgc
atcattgatctacatagataaggaggccaccatgttcaagaccatactcgttcccaccga
cggctccgaacgctcggacaaggccattgccacggcggtggactatgccaaaaacagcgg
t
>Hsero_3965
gcccgaggaggtggcgaagaacccggccagcgtcaccggcaagtacctggcgccgctgtt
gaagaagaagtcgaagtaaaaaaagaagtccggacttgcgcgcgcgcaaacgatacagat
tttgcctgcatctgtatcttgcgcgccgccggccttggcatacagtcatgcacctggccg
ccatccggcgcacaatgaaggctgtctcctccatcgggagggggctgccgtgcaaccccg
aaagtcttccatgtccaagccgctctcatccacccaggtcgccttcttccgcgaacaagg
c
>Hsero_3974
ttgcaggaagccaagtggctcatcaagaacatgcggcaacgtttcgatacaattttgcgc
gtcgcgcaggccattgtggaacgtcaaagaaacttcttttcacatggggcagtcgccatg
cgccccttgtgctacgtgaaattgctgatacactgggtctacacgagagcactatctct
cgcgtgacaactcagaaatacatgctcacaccgcatggcatgtttgagttgaagtacttc
ttcggtagccacgtcgcaactgaaaccggaggcgaagcttcctccaccgcgatacgggcg
c
>Hsero_4240
aatgagcaggatgccggcgatcagccagaggctgcgagggacagggctgaccccatgggg
atgggtggcgggcgtgggaagcgtcatctgggcggagtttgatctgggacgataagcccc
cagcttagccagtcgcggcctgcgcgtatttggctacaatgccaggatattgctaaaaac
ggtcaacccggaccctctatggccactctctacgccagcctggtcgaacacgatccggac
cgcctcgacgccgcggtcacggccttgcgcgtccacaccagccaggacgagcgcgaaacg
c
>Hsero_4703
gatgggcgtgagggccgcgtaattggcggcattgcggccaaggccggtgtcgaagtctgc
catgctggtctccttgcagcgggatgagggtggcggcctgtgcatttttttatcagccgcg
tgctatcgcgctagggtagcaccgatgccgccttgcgctttgtcatcacgacgacaaggc
tgacatgcgacaatgtttgccgtttccctccacctccgtgcccaccctatgagcagcccg
atggcgcagcttctgcagcaatacgactggttcgccgcgctggccagcgagcatcgtgcg
c

**Group II**

```
>Hsero_0151
gcggccacgcccgccgccgcggtcggcgacgctgctgccgacgaagcacagcaaagctga
tcgccgacaaggcgcatcgtctcaggcaggagtaaaaagcatggataaaaaaaccccgg
agaacagggatattctccggggccgaatgcctttcgcttgcacgatcggccccgctcagg
gagggaaggggaagcgggggtgaatgcatcacccgcagctaaggtaaagtatcttgagg
gaaagttgttcatcctttaattcaaaggaggcaattttccgaagataacgataaatagct
c
>Hsero_0253
ggcgccaccacgtcgccgcccgcctcgccaggctagcttgccgccaggaaagcccgctcg
ccaggcgcgcaaggccgcccgatttttgtttgacttttgcggcgcgatgaataatagta
cgatcgttcgaataaattctgcatgaagaatagttcatgcgtccatgaggcagcggccac
gcaagccgcgcccgtcgacacggttccttcccgtcccgcccgggggcggatagcaaagag
ataacgatgacagagacaagcaaggtgaccaccgcgcccgcccgccccaagttcctgcgc
a
>Hsero_0627
gatttcctgcagcaggccgtggaggaactggccagcgcgatccgcgccgccatgcaggcg
cgggaagggcagcgtgcgccgaaggcgccagcgcgcctgggactgagcccggtcaaggca
ggcgcgcgcgaaatgctgcatgatgcagcttgggggcggtcttgcagcgctatacggcc
gatgcggtgctgcagccgcccggcccggaagatgggctcaggccggccacggcaaggata
gtggccattggcgcgtccaccggcggaccgcaggcgctggagcgggtgctgggcaagcta
a
>Hsero_0962
tcgtgctggtgtccttcgccgccaatgccggcacctttatcggtgccaccctggtggccg
agatcttccattgagcgcaaataaaaccgcgctttgctggcccagatcaaaacatcggca
atgagcgcgactgtgcgctgacccggctgactatactggcgaaagatggccgtacggcaa
caggctgtgcggccatcttgtgtttagtcatccaggatgccctcatgcttgcccaactca
aagccgccttcaagtatttcaacgatgccgttcccttcaagctggtcccggcgctgatcg
c
>Hsero_0964
gattgtgcttgctttccagttaagcgatatgaatagagaaaaatcaagtttttgaacaat
gggtgacatggatcaatccatgcgcattcatcgattgtcgtcatgccgggcgcggaggat
agtgacatccgcaggcaggaacaacaggccgccagcgcagcacaaggccgccctgccctc
gcatcccgttgatcccccccgaggagtgcatatgacgcaaatgatgaaagcggccgtcgtc
cgcgaattcggcaagcccctgtccatcgagcaagttcccgtccccacgcctgcgccgggc
c
>Hsero_1024
atgttcatggctgcgtaacttttagttaattaccgtagactgacgcctacggggcagtgt
ctcttccatgtcttgcctggcttgccttgcgctgggtcaggctgggtgctcgcttgcatc
gacgccagcggagagcctcgctacaatctcttcccaatgctgctaaatgttttgcaaata
acatttaatggatgtaacgttacatccataactttttacttgcaaccgtttttgttaga
aacattttcccgctcaggaaaaaatcgcccatggcttccacgtccaccccttccaccaaa
g

>Hsero_1103
tggccatggcttcggcggaaatggccgaaccggccaccagggtggcgatcaacaacaggt
atttcatggaggctcctcagctagcggtgaacgacgggcctgcggcaagggtctagccgc
cgctttcgcaggcggggctattctacccggcttccccggaccgccactatctgcccggac
gtatatctgtcatagatcaaatctcctgcgcgatcgtcccccccagcgcggcggcttgtcg
cttgatatcgcgcggggatatcatgagggcccgattgattcggatccgatatcagccatg
t
>Hsero_1639
atccagttccttgcagcagagtctatctgtcttggaatgcctctcaaaaaatcacctaag
tccttgattagacgtggatcaactttccactgcttttcgccattcccatgtgatgaaagc
tttgactttatgcacgattaaactagaattcgcttgtgcaatgcatcatatttcaacaag
```

aaatgacatcggaatccacctgttcgtcgagatcggtccgtaagtgttcactgtcgcgct
gccttgttcatcaaccaactaaagagaaggatgtttcaacatgacgacctacaccgagca
a
>Hsero_1694
ggtcacagggccaacggcagcagccgcgctgcggcttgccgtgccattgagccacaaaaa
acttgtgactccatgacgcaagtcagcttactgacaaaccacgacacgccggcaaacact
gaaatatagtcgcggttacaattcaaagattttggatgcaggctcaagaccgaagaagcc
caccccggcgcagccttgcgcgggccatgagacacctggaaagctgaacaaagctgagca
ataagctgcctcctcgcaatggaggccaacaggatcagcgcatgaaaacgttttgatcgt
c
>Hsero_1710
aaaaatttccgattagaaatgatttgcccgcatcggacgcaggagactcagcgtttcggc
gcgcctagcgctagcgctatttcatattttccgcatatcaatatgagcgaaaaatgaaat
atatcaagcaaattgctttatttgcccttgactgcgccctaagatgttaaatatgagaaa
tacacgaccgacctcacctacagccatacttttataggatcaatcaaatgggacgcgccg
ccagatctcacgaaagcgacgcaccgcccagcgaccccgcaatgacttcccgggaggccg
c
>Hsero_2263
cgccgggctggtgaggcggcgcagataatcgatgggcatgacaggtccggttaggcgcga
gaacgctggcgcagcctcatcttcaagccgtcctaccccccatcgagggcggcttcttgct
gccttttccttcagcgttttctatgctgacaagcatacttcttcttgcgccctcatggtt
gccgatcagcacatacctgatgtgcgtcagccggtgcaagcaggtcttgtgttttcacct
tgccacaggaggacccattccatgagcattcccatcgatattcccggcggcgacaagctg
g
>Hsero_2511
cggtcctggaggaacaccagttcatgcccatgggcggcattggcgcgggcgagcaaggtc
tggtcgtagcggcgggcgctgaatacggcggtcttcatgcaatcattctcctggttcgcg
gcatgggatcgagatgatccatgcggcattgcaacagatcgggccggcgatggcattgac
gcaacgcagggatgggacaaacaggccgctggatgactgtccggagtccgaagctttcat
ccagctatcgaaaaaaccgatatttgcatcactcccccggccaagccgtccatctgcccc
g
>Hsero_2530
gcgcaccgattcctcgtgtggttgggcatggctcaggtgctgccgcaggcgggcgatgtc
ttcgcgcagttgttcggcttcggcactgccggcgggcagcttttccagttcggtctcgaa
ctggccgaccatgtccagcaggcgctggtggtcgaaacgcggggtttgcgcaggctcgct
catagattctccttcggtgattgaccggcgtccatcataggcgcattggcgccggtgcgg
caaaaaaagcggtcggggtttgcccggcatcaagttctcctgtttggttgcagagggacg
c
>Hsero_2983
gcgcctgacggtgctggacagtgggttgtggaaggctgtgcgccagacgcgcatggaaag
tggcgacatcgagttgttctgagtgggacaagcttgaactaggtcatttcggtccatgct
catcattaggaccgaagccaagggtgcggcataatgcgagagcgcatgagccttattgaa
gaaacagggagtaacgatgtccaaaacgattctcgcagtagatgattccgggtcactgcg
tcagatggtggtattcagtctgaaggccgcgggttacaacgtcaccgaggccgtagacgg
g
>Hsero_3055
cacaacgcgaaaccacaacgcggaaccacaacgcccggccaccgccacaccgacgaagcg
ggctacggctgagctgatagttggaccttagttgatccgcatcatgtttcgtatcagcca
agctcttctggatgtgtaggaaagagtgaaaatgtcttcatgcagcgcaacacagggctg
catcttgtggaaccgggaagtccaccagatgcaatgcttgcgcgacatttcgtcgcaccc
tgccagtgccctgcagaggacctcgccccgcgcatgcgcgccgggcctgggtcattctca
t
>Hsero_3146
ggttagacgcatgaattccgctccagaaacaagtgaaccgattggccctgaattcccgaa
tatttgagtcaagtatagcaaacccgagtaattctgtcaggtacaagcaccgacccagat
catcattccttcatattgcggtgaaacttgtctgacctcctccgcaaattgcgctaatct

cacgctttgggccggcatgttcaggattggccacttatcgatctggtaagatgtgccgaa
cttttccatcaggcgtcatgcggactgaccgaacaaccacaagcccggtccgcgccgcca
c
>Hsero_3223
tgcgcgccgccatccagtggccgcagatccgcggctgggcgccgctggacctgctggcgg
cgggtatcccgcccatggggcgtccctgagccatgatccagcgcagctgtctgcgctatc
ccggttcaccggagctgacaaaatggtgtaaattggaagctgacccacggaccggtcggc
ccctcaagcgcgccgccggcttcccgcaagacaggacaggaggcacatgacgcatctcaa
agacttgccgttctccacgttgcagttctatgcaaccgcgccctatccgtgcagttatct
g
>Hsero_3327
cggatgcgatgcaaatgcaacggcagcccgccggatgtggctgccagcggcgccggtggg
aaatttctttttgacgctctgcgcaaaacgtcctaatattgaccgcatgatcgccaacgc
cacatttttacgacgccgcagccaacctgagaaaggtttgctgctgcgcgcgtgaagtga
aacgaatcgcgaagccacaggcaaaccctgtggcttttttttattcgccattttgggtct
gtcgtgcgcctgccgcctagcgcttagtgctcatgtgtcagcaacggtctgcaccaccct
c
>Hsero_3370
aacagggaggcgcagcacaggacgaagcatgatggaacctggaatgagggtccatcatta
taggggaagccgtctgcgccttgcccggggggagcccgacgccgaccgcttgcgccaggtc
aggaatgatcacttgcggacatgagcatgtcactgcgatatgcgacaatcttcgcctact
gtcagggaggccccatcccgacggcgaacaggacccaagaaaaataccaccatgccctcc
agttcccctccgtcgcacaagaccgcgccgctgcatgagcggctggcgctctggcgcgag
c
>Hsero_3472
aaaggctggcaagagcaccgacaggtatagctcatgcgcaagacgctggaagaatttcta
ctggcctaccgatcgacaccgaaagtcgaatgcatacggttgatgtaagtcatcctatgg
ttgatgcagtgaaactgtatcttccaagtggtgtagattctagaatcagagaatttctta
acacggttcgatattacgatgcgctccaagcgtctgaggcagcgtcacctcttagactat
tgatttacggagcaccaggcacggggaaaactcagactgcgcgttggattgcgggtgaac
t
>Hsero_3645
catgaattcaacgaggcgccacagaagatcgtgcgtttcatgtcacagacgggtggctag
ccggactgcctgttgcagtttcccaggctttctgctgctttttaagaaacattgacttg
gcttccatacatgctggcaacgatgggcatagactgcctgtcgttcccgccccgagtacc
ctcatggaccctgccgccgctcccttcgcgcccttgcccccgcacaagcgcctgaccttc
cgcatcctgctgatgaccctcatcggcctgggcctgaccatgacggccatcggctatacc
c
>Hsero_4274
ctatggttatggctaccagcttgaacggctggactgaggccccgcagggtgttgcagcag
cgccatgacgcgggggcaacactgatccatatcagggaccacgatgctttctcccgcac
cggccccaaccggaaatcgggcaaagagtataattgttcccaaaaccggggctgaatctg
tccatttttccgcccgaatctgcaccatccctgctcaacggccctgctgccgcgtactcc
atgcatttgcttgccgtcggactcaaccacactaccgcgccggtctcgctgcgcgagaaa
g
>Hsero_4704
gcgcacgatgctcgctggccagcgcggcgaaccagtcgtattgctgcagaagctgcgcca
tcgggctgctcatagggtgggcacggaggtggagggaaacggcaaacattgtcgcatgtc
agccttgtcgtcgtgatgacaaagcgcaaggcggcatcggtgctaccctagcgcgatagc
acgcggctgataaaaaatgcacaggccgccaccctcatcccgctgcaaggagaccagcat
ggcagacttcgacaccggccttggccgcaatgccgccaattacgcggccctcacgcccat
c

**Group III**

>Hsero_0005
aaagcgactatgcagactttatttcgcgtttgccacggtcattaaaatcggttggtcttg
gcgctaggctgacaaaaaaagagaggtttgcgttgttagctagacttaactcaagttacg
tgatttagctgatcagtaattcaagctacaatggccccaataacaattttctcaggggc
tagaatgtatgttccatatctatatgggcagaagtttgaattgcaggcgcttcgcgaatt
agttaccaagttagccaccaagaggcgtgttaatccgttaattgacccggtaactagcaa
a
>Hsero_0153
gcgacatggtgggggaaatccgaaagaggagggaaaaacgcggagaagctattgtacaa
tggcccgaaacccaacgcctcgttgacgccgggcaagtaaaaaatatggcggccattgga
gacaggtcaaacaacaggcatttggtttaggggcagctagccgccgaacgggcaaaatct
ctcaattccacaataagtattagggtgacgaatgaaccgtgccttttccccactgttgta
ctccgtttttgctgccttcctggcagtggccagcacagcccacgcggctgatgacaagaa
a
>Hsero_0998
atttatttctgaatatatggaatatgtggaatgtgaggtaaggcaaaacgaacaaattca
acaccagcaattggtaccaaattgatactgattaactaccactcaaattaatattgatta
actccaactcaaatgacagaatgcccgcagatgttggtgcaaagtgactgctgtagtctt
atcaatcaaaacaaaacacacacttgcaaattggggggattcaaaatgcattatccgttcc
ggaaagcttccgcattactggcattggcttcaggtctgatgactgccgcatgggcgccaa
a
>Hsero_1104
acatggctgatatcggatccgaatcaatcgggccctcatgatatccccgcgcgatatcaa
gcgacaagccgccgcgctgggggacgatcgcgcaggagatttgatctatgacagatata
cgtccgggcagatagtggcggtccggggaagccgggtagaatagccccgcctgcgaaagc
ggcggctagacccttgccgcaggcccgtcgttcaccgctagctgaggagcctccatgaaa
tacctgttgttgatcgccaccctggtggccggttcggccatttccgccgaagccatggcc
a
>Hsero_1175
cagagcgatacctacgccaagcgcgacgaagtgatggcctttttgaacccgacacggcg
cagctgctccaagcctctgcctgatttgctgccggtcaagcttggccgctgcgacggttg
cctagccgaaagcaacacgttaatctacgaaatgctttcgccttgaattgacaaaattgc
aattaacaaaaacgcagatgcgagcccgattcattttctcgatatttcccgctactggc
tggtgctgttgaccgccctgatgctgtcggcgccggtgcaggcgcgcggacctgcgccgg
g
>Hsero_1383
aaattccgggccgaattcaagaccgtgatgtcaaaaaagcccaaggccagtcgcgataag
tcgtcggaaatctttctactagggaaaatcctgaaaaatcctcgcagctaaatcggttaa
agttgatgcgaaacagcgtcagaagctcggtttgagcgcaaaatctgccagatttctctt
cagatgctgcgttttcccgacttgttttgcctgatttcagcctcatatcgattttcagga
atcgtctgattgcctctaccggctgtccaggactgctgatggcgaaatgacaagcctgcc
t
>Hsero_1545
ccggatgggagcgagatccgattctgctgccgcgcaaaagcgcaacggcaagccgcggat
ttgggggcgtatttacacgcccttggccacaatccggcgagctcccttcatcctgtgcc
agatcaaatgtgagcagatccgtgcctgcgggaaacacgatgctgtgccaaaatgcataa
atgttgtcctgaacataaggggatatgaaggtcttcttcaatgaagcccttgtggcaag
acccgcccagccctgatgcaagcggcctcccgtgctggaagggcgctgcttgtgtaagga
c
>Hsero_1829
actgatcgccaccgcgccggggccagctacagtggccccggcgcggcgtggatggcctgg
tcgtcaataaccaacccatcagtcagtaacctgacgcacatcaaggccgcatcggcgcg
gatcttcataatgtggacacaaaagacgaacttcgtcgccacattatctgcccacgccga
tgaccaccacgcgcccgctgctgacgagaacacgcctgctgctggcggtcttgcccttgt

gcctgaccgcttgccagagcctggcgccgcattaccaacgtcccgctgcaccggtggcgg
c
>Hsero_2110
cttgcatctgctgggctccatcgtgctgtgtgttgccgggtttgcaagctatcgcgccct
cgctggatgagcactccgcgtgacctgatgcagatcaaaacagacggcacgatcattgct
tgaaaatcgcaggcgcaggcccaactttcatcttattgaaaaattggagcgaaccatgcg
tctcgacaaactcaccaccaaattgcaggaagcgctggccgatgcgcaatcccaggccgt
cggccacgacaatcaatacatcgaacccgtccacctcatcctggcgctgctgaaccagga
g
>Hsero_2290
accgcttgcctgcgggcaggtcgccgtcctttcggcgcgcctggtttgatccagcgcaag
cccgcaccagttctgctttagggctggtggcgcttgatgcatgtcaagtcgtctcagga
gtcgggggcttagtatgcagtcatacgatttctgtcacccgagaggtcatcatgttcaag
aacatcctgctggcaaccgacggttcccgtctgtgcaacgagtcggtcaaggcggccatt
gccctggccaagagctgtggcagcagcctggtcggcttgtcggtatcgggcaatctgcgc
g
>Hsero_2488
gaggccggggatcaaagtccacaagcataccccagccagcctgatcgcatcattgctgcg
ctggaacaggcggcgcggcggacaagccggctgtgcttgacatgtgtcaattgcgcccgg
ttttgcgctggacgagggccgcacgcaggcctagtctgaagccatggcatcccatcgcgg
acgcctttccatcccaacaccccgaggagctaccatgagctacaagtcgatcctggtcca
tgtcgaccagtcacgtcacgctgcccagcgcatccgcatcgccgccgaactggcgcgcgc
c
>Hsero_2510
tgagcctgcatggcggcgccgagtgcggtgccatgcagcaagttgttccggacaatctgc
gcgttttgaacgaagacctgcgccggatcaggcaatcattgatcttcgacaactttcggc
ggcgcagcaaatggtaggctgtgcactccccctcctcccgccgaaagacgaccatgaccg
agaaactgagcgcccagcgccacacccccgatgagttgagccaggaactggaagcactgg
acatggaaatcgtgcgccacgccgtgatctgccaggtgcggctgtttgatccgggcatcc
t
>Hsero_2530
gcacagggtgcgccgcgcgcaaaaacagccgccagtgtaacaaagcacgggcggctgccg
gcgctggaacttagtgcgtccctctgcaaccaaacaggagaacttgatgccgggcaaacc
ccgaccgctttttttgccgcaccggcgccaatgcgcctatgatggacgccggtcaatcac
cgaaggagaatctatgagcgagcctgcgcaaaccccgcgtttcgaccaccagcgcctgct
ggacatggtcggccagttcgagaccgaactggaaaagctgcccgccggcagtgccgaagc
c
>Hsero_2915
cttgagcgatagttacgtattcttaaagttggtcatgcgcttctcatcgccgccgatgtt
gctgtcctgcaagccgcggcaggtggaacatgatccgcatcaagccaaggccagatgcgg
agtttctaagtttttgagcgacatataatttcgatgcaccttaatggaacacacctgcac
atcatctgggatgtgcgcatcggacgtcgaacatgaccatcgctaagaaactccatctcc
tgaccctcgcggtcatcctcggtctggccagcctgtcattgctgggcatctatcaaagca
g
>Hsero_3148
cctgatcgcctcggggccggagttggctctccgccatgttccggccctgagcggacgaag
ggacttgcctttccgataagtctgtttttgtgcaatctttcatatgcttgatccaaaaca
ggcaggtcacttcctgaccgctgcacaatggcgcaaggtcaggcgctgaccgccctatcc
gggaaataggggtggttttggtcagtttgccgtcttatgggggagacgggggctgatgta
tcgtcggggcaataattcatttcttgtgttgtacaaaaactaagcggagagagggaatgc
g
>Hsero_3197
atcgaatttgtgtggtatttcccacctcaatctggcggtcttccggccttccgtagcgtt
gccggccgtttagttccgacatgtttgatctgggacaagaaatgccgtctgattcgggaa
tactgtgcttcctatattgttccgcgcacttaaaatcgagtcattcgattgccacatgtc
cgccgggaacatcgtatgaaccagtcttgccactccactccgatcaccacgccggctaac

gccgctgccccccctgacccactgctcgtcgctgaccgcggcccgcgaggctgccgccagc
t
>Hsero_4049
tcctgcccgccttgaaaagtaaatcggacaggcgctgacgccgcagcgcaagataaccga
caaaaagagcagattgacaatgggttgcgctgcgtcaaatgttcattttcacaagtctgt
accatacccgccgttagctacaaaaccctattcagcttatatataatcaaaaagttgcat
gagggtcacttagaaggtccacgctaacttacatttggagattggggtctttatgactga
cgagaatcaggtcgacgcgagtcgacgaggtttgcttgttgcgacttgtgcggccggagg
t
>Hsero_4190
cttcaggtcgggctttcggatcttggtggcccggggcggaatcgaaccaccgacacaagg
attttcaatcctctgctctaccaactgagctaccaggccaagacggacaagtatagacgc
tttaaaaaattatgcaagtcctttttgaaaaatagttttcgccaccccgtttataatcgc
cagcatgactacccacgcacaccccaattcctcttcgtcggccggggccggcactgatat
ctgcatcgtcggcgacggcgcggtcggcaagacggcggcgctggggctggcgcaggctgg
c

**Supplementary File S2**

List of motifs found in the MEME search

**Group I**

| Locus Tag | Position | p-value | Upstream | Motif | Dowstream |
|---|---|---|---|---|---|
| Hsero_0239 | 100 | 1.61E-05 | tggcgtccgc | TTGATTTACCGAAA | attcaccgta |
| Hsero_0260 | 28 | 2.16E-05 | ccgggcgcga | TTGCGATGCATCAA | cgcggtattg |
| Hsero_0262 | 261 | 3.22E-06 | caataccgcg | TTGATGCATCGCAA | tcgcgcccgg |
| Hsero_0371 | 87 | 5.81E-05 | gcgagcttct | CTGATGCCGCGCAA | ggcgacgcct |
| Hsero_0624 | 114 | 2.38E-05 | atcgaccggc | TTGGAGCAGATCAA | caatgccgtg |
| Hsero_0660 | 98 | 1.61E-05 | ttgcgcggcg | TTGACCCAGGTCAG | cgccaacagg |
| Hsero_0835 | 129 | 6.92E-07 | tccaagtatt | TTGATGCAGCGCAA | taagctgacg |
| Hsero_1002 | 129 | 4.41E-07 | aagagtgagc | TTGATATTGATCAA | atggattgtt |
| Hsero_1165 | 119 | 1.45E-05 | aagacggcat | TTGATCGAGTGCAA | agaattgtct |
| Hsero_1250 | 89 | 7.91E-06 | ggaatggatt | TTGCTAAACCTCAA | aagcagcact |
| Hsero_1337 | 151 | 3.45E-05 | aggcctgttt | TTTGCATAGATCAA | aacgtggtgt |
| Hsero_1446 | 107 | 6.28E-06 | ttttgggcgc | TTGATATGGGTCAG | agcaggatga |
| Hsero_1552 | 129 | 2.92E-08 | cagtgccggc | TTGATGCACATCAA | gaacgataac |
| Hsero_1880 | 156 | 3.52E-04 | tcatgacgct | CTTATGCAAATCGA | agaccccaac |
| Hsero_1957 | 81 | 4.89E-05 | gcgacaaaaa | TTCATGAAAATCAA | aatgatgtga |
| Hsero_1976 | 104 | 1.77E-05 | atgggccggg | TTGTCCTCGATCAA | tgcttgcgca |
| Hsero_2144 | 259 | 2.87E-04 | tgcagccttg | CTGTACCAGGGCAA | gctgctgtcg |
| Hsero_2379 | 121 | 6.92E-07 | tcggagattt | TTGATACCCATCAA | agattcacat |
| Hsero_2384 | 114 | 1.45E-05 | ccaattccct | TTGACCTGGATCAG | agggaagggc |
| Hsero_2403 | 179 | 3.22E-06 | tatcgcgcca | CTGATTTAGGTCAA | agtgcgcagt |
| Hsero_2404 | 57 | 1.49E-06 | gctggcaaaa | CTGATGCAGGTCAA | attcagggcc |
| Hsero_2458 | 105 | 1.99E-06 | cgagcggtgc | TTGATGTGCCGCAA | gccaggcctg |
| Hsero_2909 | 86 | 1.11E-04 | aaattcccaa | TTGATCAAAGAAAA | gatttcttct |
| Hsero_2999 | 206 | 2.68E-04 | tgactgttcg | GTGATCGACATCAT | gaagacgacg |
| Hsero_3072 | 150 | 7.91E-06 | tgctcacgta | TTGACATATCGCAA | gcatcgccgg |
| Hsero_3111 | 94 | 2.85E-06 | ccacgctgcc | TTGATCCGCCGCAA | gcgcagcgcg |
| Hsero_3194 | 93 | 5.16E-07 | atggccgcgc | TTGATCGGCATCAA | agccggacag |
| Hsero_3203 | 100 | 7.92E-08 | tctccccatt | TTGATACACGTCAA | ggctgccgga |
| Hsero_3207 | 107 | 1.91E-08 | gtcaggcctt | TTGATACACATCAA | ggtgtttttg |
| Hsero_3246 | 78 | 6.60E-04 | tttatcctgc | TTGTCGGTGCTAAA | tccttgtggc |
| Hsero_3273 | 139 | 2.33E-04 | actccgtgat | TATACCTGCATCAA | acttggtggc |
| Hsero_3488 | 124 | 5.01E-06 | actcggattt | TTGATATGGATTAA | atagctcgca |
| Hsero_3532 | 161 | 5.33E-05 | cgagtcagtt | TTGCTTGATGTCAA | gatttctgca |

| | | | | | |
|---|---|---|---|---|---|
| Hsero_3853 | 97 | 2.62E-05 | cccttgtcgt | TTGGGCTATATCAA | aagcgggcag |
| Hsero_3886 | 185 | 9.82E-06 | gggcgcatca | TTGATCTACATAGA | taaggaggcc |
| Hsero_3965 | 96 | 5.50E-04 | aagtccggac | TTGCGCGCGCGCAA | acgatacaga |
| Hsero_3974 | 74 | 4.89E-05 | gcgcaggcca | TTGTGGAACGTCAA | agaaacttct |
| Hsero_4240 | 99 | 5.81E-05 | tgggcggagt | TTGATCTGGGACGA | taagccccca |
| Hsero_4703 | 180 | 1.11E-04 | gacgacaagg | CTGACATGCGACAA | tgtttgccgt |

**Group II**

| Locus Tag | Position | p-value | Upstream | Motif | Dowstream |
|---|---|---|---|---|---|
| Hsero_0151 | 41 | 2.05e-04 | gctgctgccg | ACGAAGCACAGCAA | agctgatcgc |
| Hsero_0627 | 103 | 5.87e-05 | gcgcctggga | CTGAGCCCGGTCAA | ggcaggcgcg |
| Hsero_0962 | 97 | 1.10e-05 | ccgcgctttg | CTGGCCCAGATCAA | aacatcggca |
| Hsero_0964 | 211 | 2.62e-05 | aggagtgcat | ATGACGCAAATGAT | gaaagcggcc |
| Hsero_1024 | 87 | 5.87e-05 | ctggcttgcc | TTGCGCTGGGTCAG | gctgggtgct |
| Hsero_1103 | 187 | 1.81e-05 | ggacgtatat | CTGTCATAGATCAA | atctcctgcg |
| Hsero_1639 | 69 | 2.62e-05 | agtccttgat | TAGACGTGGATCAA | ctttccactg |
| Hsero_1694 | 73 | 1.78e-06 | ttgtgactcc | ATGACGCAAGTCAG | cttactgaca |
| Hsero_1710 | 114 | 1.10e-05 | tgagcgaaaa | ATGAAATATATCAA | gcaaattgct |
| Hsero_2263 | 197 | 1.99e-05 | cagcacatac | CTGATGTGCGTCAG | ccggtgcaag |
| Hsero_2511 | 176 | 2.86e-05 | ggcgatggca | TTGACGCAACGCAG | ggatgggaca |
| Hsero_2529 | 260 | 1.10e-04 | cggtcggggt | TTGCCCGGCATCAA | gttctcctgt |
| Hsero_2983 | 94 | 1.01e-06 | tgggacaagc | TTGAACTAGGTCAT | ttcggtccat |
| Hsero_3055 | 92 | 3.72e-06 | tggaccttag | TTGATCCGCATCAT | gtttcgtatc |
| Hsero_3146 | 110 | 8.92e-06 | ggtacaagca | CCGACCCAGATCAT | cattccttca |
| Hsero_3223 | 227 | 3.72e-06 | caggaggcac | ATGACGCATCTCAA | agacttgccg |
| Hsero_3370 | 109 | 1.64e-05 | cgccgaccgc | TTGCGCCAGGTCAG | gaatgatcac |
| Hsero_3472 | 100 | 1.35e-06 | atgcatacgg | TTGATGTAAGTCAT | cctatggttg |
| Hsero_3645 | 232 | 2.86e-04 | gcacaagcgc | CTGACCTTCCGCAT | cctgctgatg |
| Hsero_4274 | 83 | 1.55e-06 | gggggcaaca | CTGATCCATATCAG | ggaccacgat |
| Hsero_4704 | 109 | 7.22e-06 | acggcaaaca | TTGTCGCATGTCAG | ccttgtcgtc |

**Group III**

| Locus Tag | Position | p-value | Upstream | Motif | Dowstream |
|---|---|---|---|---|---|
| Hsero_0005 | 189 | 5.12e-5 | gctagaatgt | ATGTTCCATATCTA | tatgggcaga |
| Hsero_0153 | 83 | 9.80e-6 | caacgcctcg | TTGACGCCGGGCAA | gtaaaaaata |
| Hsero_0998 | 82 | 1.35e-5 | tggtaccaaa | TTGATACTGATTAA | ctaccactca |
| Hsero_1104 | 102 | 3.35e-6 | cgcaggagat | TTGATCTATGACAG | atatacgtcc |
| Hsero_1175 | 86 | 2.45e-6 | tctgcctgat | TTGCTGCCGGTCAA | gcttggccgc |
| Hsero_1383 | 123 | 6.43e-6 | tcggttaaag | TTGATGCGAAACAG | cgtcagaagc |
| Hsero_1545 | 114 | 1.95e-5 | tcccttcatc | CTGTGCCAGATCAA | atgtgagcag |
| Hsero_1829 | 91 | 5.34e-6 | agtcagtaac | CTGACGCACATCAA | gggccgcatc |
| Hsero_2110 | 85 | 2.77e-7 | tccgcgtgac | CTGATGCAGATCAA | aacagacggc |
| Hsero_2290 | 95 | 2.73e-8 | ctggtggcgc | TTGATGCATGTCAA | gtcgtctcag |
| Hsero_2488 | 97 | 8.26e-6 | ccggctgtgc | TTGACATGTGTCAA | ttgcgcccgg |
| Hsero_2510 | 99 | 2.45e-6 | caggcaatca | TTGATCTTCGACAA | ctttcggcgg |
| Hsero_2530 | 104 | 1.78e-6 | acaggagaac | TTGATGCCGGGCAA | accccgaccg |
| Hsero_2915 | 90 | 1.78e-6 | caggtggaac | ATGATCCGCATCAA | gccaaggcca |
| Hsero_3148 | 108 | 3.71e-6 | tttcatatgc | TTGATCCAAAACAG | gcaggtcact |
| Hsero_3197 | 85 | 3.89e-7 | tccgacatgt | TTGATCTGGGACAA | gaaatgccgt |
| Hsero_4049 | 85 | 2.72e-5 | tgacaatggg | TTGCGCTGCGTCAA | atgttcattt |

## Supplementary Table S1

| Strains | Description | Source |
|---|---|---|
| *H. seropedicae* | | |
| SmR1 | Z78 but $Sm^R$ 100µg/mL, Nif+ | (4) |
| Δ1 | SmR1 derived with a 774 bp deletion at the *fnr1* gene | This study |
| Δ2 | SmR1 derived with a 723 bp deletion at the *fnr2* gene | This study |
| Δ3 | SmR1 derived with a 687 bp deletion at the *fnr3* gene | This study |
| Δ13 | SmR1 derived with double deletion at *fnr1* and *fnr3* genes | This study |
| Δ21 | SmR1 derived with double deletion at *fnr1* and *fnr2* genes | This study |
| Δ23 | SmR1 derived with double deletion at *fnr2* and *fnr3* genes | This study |
| Δ231 | SmR1 derived with trilple deletion at *fnr1, fnr2* and *fnr3* genes | This study |
| Fnr1-3xFlag | SmR1 derived expressing a Fnr1-3xFlag protein from the chromosome | This study |
| Fnr3-3xFlag | SmR1 derived expressing a Fnr3-3xFlag protein from the chromosome | This study |
| $Fnr1^{AR3->3}$-3xFlag | SmR1 derived expressing a $Fnr1^{AR3->3}$-3xFlag protein from the chromosome | This study |
| $Fnr3^{AR3->1}$-3xFlag | SmR1 derived expressing a $Fnr3^{AR3->1}$-3xFlag protein from the chromosome | This study |
| *Δfnr1*/Fnr3-3xFlag | Derived from *fnr1*deletion mutant, expressing a Fnr3-3xFlag protein | This study |
| *Δfnr3*/Fnr1-3xFlag | Derived from *fnr3*deletion mutant, expressing a Fnr1-3xFlag protein | This study |
| *E. coli* | | |
| DH5α | Cloning strain | Invitrogen |
| S17.1 | Conjugation strain | (5) |
| BTH101 | BACTH reporter strain | (6) |
| **Plasmids** | **Description** | **Source** |
| pK18mobsacB | $Km^R$, Mob. Suicide vector for gene replacement. *sacB* gene for counter selection | (7) |

| | | |
|---|---|---|
| pJQ200SK | $Gm^R$ , Mob. Suicide vector for gene replacement. *sacB* gene for counter selection. MCS from pBluescriptSK. | (8) |
| pPW452 | $Tc^R$.Transcriptional fusion vector dervided from pMP220, but with inverted cloning site. | (9) |
| pUT18C | $Cb^R$. BACTH vector - T18 fragment for N-terminal fusions | (10) |
| pUT18 | $Cb^R$. BACTH vector - T18 fragment for C-terminal fusions | (10) |
| pT25 | $Cm^R$. BACTH vector - T25 fragment for N-terminal fusions | (11) |
| pKNT25 | $Km^R$. BACTH vector - T25 fragment for C-terminal fusions | (12) |
| pKT25-zip | $Km^R$. N-terminal fusion of leucine ziper domain and the T25 fragment of the adenylate cyclase. Positive control for BACTH assays | (10) |
| pUT18-zip | $Cb^R$. N-terminal fusion of leucine ziper domain and the T18 fragment of the adenylate cyclase. Positive control for BACTH assays | (10) |
| pJET1.2/blunt | $Cb^R$. Blunt cloning vector. | Thermo Scientific |
| pBlueScript KS II + | $Cb^R$.  Cloning vector. | (13) |
| pMBB1D | $Cb^R$, $Cm^R$. pSUP202 dervived vector containing a copy of *fnr1* gene (deleted by 279 bp) flanked by 198 bp and 245 bp of upstream and downstream regions, respectively. | (14) |
| pMBB2D | $Cb^R$, $Cm^R$. pSUP202 dervived vector containing a copy of *fnr2* gene (deleted by 276 bp) flanked by 333 bp and 357 bp of upstream and downstream regions, respectively. | (14) |
| pMBB3D | $Cb^R$, $Cm^R$. pSUP202 dervived vector containing a copy of *fnr3* gene (deleted by 267 bp) flanked by 315 bp of both upstream and downstream regions. | (14) |

| | | |
|---|---|---|
| pMB1231 | Km$^R$. pK18mobsacB derived vector containing a copy of *fnr1* gene (deleted by 774 bp) flanked by 198 bp and 245 bp of upstream and downstream regions, respectively. | This study |
| pMB1232 | Km$^R$. pK18mobsacB derived vector containing a copy of *fnr2* gene (deleted by 723 bp) flanked by 333 bp and 357 bp of upstream and downstream regions, respectively. | This study |
| pMB1233 | Km$^R$. pK18mobsacB derived vector containing a copy of *fnr3* gene (deleted by 687 bp) flanked by 315 bp of both upstream and downstream regions. | This study |
| pMB1300 | Cb$^R$. pUC57-Simple derived vector containing the coding region for the 3xFlag peptide flanked by BamHI, KpnI and XhoI sites at 5` primer end and HindIII, SalI and XmaI at 3` primer end (Genscript Corporation). | This study |
| pMB1301 | Cb$^R$. pMB1300 derived vector containing the *fnr1* gene cloned at the 5` end of the 3xFlag coding region (BamHI/ XhoI fragment) plus 1026 bp of *fnr1* downstream region cloned at the 3` end of the 3xFlag (HindIII/XmaI). | This study |
| pMB1303 | Cb$^R$. pMB1300 derived vector containing the *fnr3* gene cloned at the 5` end of the 3xFlag coding region (BamHI/ XhoI fragment) plus 872 bp of *fnr3* downstream region cloned at the 3` end of the 3xFlag (HindIII/XmaI). | This study |
| pMB1304 | Cb$^R$. M13F/M13R PCR product from pMB1303 cloned into pJET1.2/blunt. The selected orientation was the one where the 5` end of *fnr1* is near the XbaI site from the vector backbone. | This study |
| pMB1305 | Gm$^R$. BamHI/XmaI fragmenf from pMB1301 cloned into pJQ200SK. | This study |
| pMB1307 | Gm$^R$. XbaI/NotI fragmenf from pMB1304 cloned into pJQ200SK. | This study |
| pMB1201 | Derived from pPW452. Tc$^R$. *pfnr1::lacZ* fusion. | This study |
| pMB1607 | Cb$^R$. *fnr1* gene promoter with altered Fnr DNA binding motif *pfnr1**. The mutagenizaed promoter was generated by overlapping PCR and then cloned into EcoRV site of pBlueScript KS II + | This study |
| pMB1608 | Tc$^R$. *Pfnr1*::lacZ* fusion. PstI/BglII fragment from the pMB1607 cloned into pPW452 linearized with the same enzymes. | This study |

| | | |
|---|---|---|
| pMB1601 | Cb$^R$. *fnr1*$^{AR3\text{->}3\text{-}3xFlag}$ gene generated by overlaping PCR (pMB1301 used as template) cloned into EcoRV site of pBlueScript KS II + | This study |
| pMB1604 | Cb$^R$. *fnr3*$^{AR3\text{->}1\text{-}3xFlag}$ gene generated by overlaping PCR (pMB1304 used as template) cloned into EcoRV site of pBlueScript KS II + | This study |
| pMB1609 | Cb$^R$. BamHI/HindIII fragment from pMB1601 (*fnr*$^{1AR3\text{->}3\text{-}3xFlag}$) cloned into the vector pMB1301 linearized with BamHI/HindIII | This study |
| pMB1612 | Cb$^R$. HindIII/XhoI fragment from pMB1304 (downstream region of *fnr3* gene) cloned into the vector pMB1604 (*fnr3*$^{AR3\text{->}1\text{-}3xFlag}$) linearized with HindIII/SalI | This study |
| pMB1615 | Km$^R$. BamHI/SmaI fragment from pMB1609 (*fnr1*$^{AR3\text{->}3\text{-}3xFlag}$ + downstream *fnr1*) cloned into the vector pK18mobsacBKm linearized with the same enzymes | This study |
| pMB1618 | Km$^R$. EcoRI/SmaI fragment from pMB1612 (*fnr3*$^{AR3\text{->}1\text{-}3xFlag}$ + downstream *fnr3*) cloned into the vector pK18mobsacBKm linearized with the same enzymes | This study |
| pMB1701 | Cm$^R$. *fnr1*gene cloned into BamHI/KpnI sites of pT25 BACTH vector. Expresses a T25-Fnr1 fusion protein. | This study |
| pMB1702 | Cm$^R$. *fnr2*gene cloned into BamHI/KpnI sites of pT25 BACTH vector. Expresses a T25-Fnr2 fusion protein. | This study |
| pMB1703 | Cm$^R$. *fnr3*gene cloned into BamHI/KpnI sites of pT25 BACTH vector. Expresses a T25-Fnr3 fusion protein. | This study |
| pMB1704 | Km$^R$. *fnr1* gene cloned into BamHI/KpnI sites of pKNT25 BACTH vector. Expresses a Fnr1-T25 fusion protein. | This study |
| pMB1705 | Km$^R$. *fnr2* gene cloned into BamHI/KpnI sites of pKNT25 BACTH vector. Expresses a Fnr2-T25 fusion protein. | This study |
| pMB1706 | Km$^R$. *fnr3* gene cloned into BamHI/KpnI sites of pKNT25 BACTH vector. Expresses a Fnr3-T25 fusion protein. | This study |
| pMB1707 | Cb$^R$. *fnr1*gene cloned into BamHI/KpnI sites of pUT18C BACTH vector. Expresses T18-Fnr1 fusion protein. | This study |

| | | |
|---|---|---|
| pMB1708 | Cb$^{R}$. *fnr2*gene cloned into BamHI/KpnI sites of pUT18C BACTH vector. Expresses T18-Fnr1 fusion protein. | This study |
| pMB1709 | Cb$^{R}$. *fnr3*gene cloned into BamHI/KpnI sites of pUT18C BACTH vector. Expresses T18-Fnr1 fusion protein. | This study |
| pMB1710 | Cb$^{R}$. *fnr1* gene cloned into BamHI/KpnI sites of pUT18 BACTH vector. Expresses a Fnr1-T18 fusion protein. | This study |
| pMB1711 | Cb$^{R}$. *fnr2* gene cloned into BamHI/KpnI sites of pUT18 BACTH vector. Expresses a Fnr2-T18 fusion protein. | This study |
| pMB1712 | Cb$^{R}$. *fnr3* gene cloned into BamHI/KpnI sites of pUT18 BACTH vector. Expresses a Fnr3-T18 fusion protein. | This study |
| pMB1713 | Cm$^{R}$. *fnr1-I171A* gene cloned into BamHI/KpnI sites of pT25 BACTH vector. Expresses a T25-Fnr1-I171A fusion protein. | This study |
| pMB1714 | Cm$^{R}$. *fnr1-E174A* gene cloned into BamHI/KpnI sites of pT25 BACTH vector. Expresses a T25-Fnr1-E174A fusion protein. | This study |
| pMB1715 | Cm$^{R}$. *fnr3-I156A* gene cloned into BamHI/KpnI sites of pT25 BACTH vector. Expresses a T25-Fnr3-I156A fusion protein. | This study |
| pMB1716 | Cm$^{R}$. *fnr3-E159A* gene cloned into BamHI/KpnI sites of pT25 BACTH vector. Expresses a T25-Fnr3-I156A fusion protein. | This study |
| pMB1717 | Cb$^{R}$. *fnr1-I171A* gene cloned into BamHI/KpnI sites of pUT18 BACTH vector. Expresses a Fnr1-I171A-T18 fusion protein. | This study |
| pMB1718 | Cb$^{R}$. *fnr1-E174A* gene cloned into BamHI/KpnI sites of pUT18 BACTH vector. Expresses a Fnr1-E174A-T18 fusion protein. | This study |
| pMB1719 | Cb$^{R}$. *fnr3-I156A* gene cloned into BamHI/KpnI sites of pUT18 BACTH vector. Expresses a Fnr1-I156A-T18 fusion protein. | This study |
| pMB1720 | Cb$^{R}$. *fnr3-E159A* gene cloned into BamHI/KpnI sites of pUT18 BACTH vector. Expresses a Fnr1-E159A-T18 fusion protein. | This study |

## Supplementary Table S2

| Primer ID | RS | Sequence (5'-->3') | Source | Application |
|---|---|---|---|---|
| 1DA+ | HindIII | TGCATCAAGCTTGTGTGGTA | (14) | PCR amplification of the upstream region of the *fnr1* for gene deletion construct |
| CHR1DA- | XhoI | TATGAAGATCTCGAGGGTGATCGGAGT | This study | |
| CHF1DB+ | XhoI | CTCGAGATCTTCATAACCGCGACG | This study | PCR amplification of the downstream region of the *fnr1* for gene deletion construct |
| 1DB- | BamHI | TCTTTTGGATCCTCAACCCG | (14) | |
| 2DA+ | HindIII | GGAACAAAGCTTTCAGCAGC | (14) | PCR amplification of the upstream region of the *fnr2* for gene deletion construct |
| CHR2DA- | XhoI | TATGAAGATCTCGAGCAAACGCTTACT | This study | |
| CHF2DB+ | XhoI | CTCGAGATCTTCATAATGAATTTGCCG | This study | PCR amplification of the downstream region of the *fnr2* for gene deletion construct |
| 2DB- | BamHI | AGGTTGGGATCCTGGTGGAAG | (14) | |
| 3DA+ | HindIII | ACTGGAAAGCTTGGCCTATG | (14) | PCR amplification of the upstream region of the *fnr3* for gene deletion construct |
| CHR3DA- | XhoI | TATGAAGATCTCGAGATGCAGGTTGAC | This study | |
| CHF3DB+ | XhoI | CTCGAGATCTTCATACTGGGCAACGAT | This study | PCR amplification of the downstream region of the *fnr3* for gene deletion construct |
| 3DB- | BamHI | GTATAGCCCGGATCCAGTTCG | (14) | |
| 1F | None | ACCAGTTGTGCCTGCCCA | (14) | Confirmation of *fnr1* gene deletion |
| 1R | None | CTGCTTGCGGAACTTGGA | (14) | |
| 2F | None | CAATGACCTGAGTAAGCGT | (14) | Confirmation of *fnr2* gene deletion |
| 2R | None | GAACTTGCTGATGAGGCG | (14) | |
| 3F | None | TTCCACCCACAAGCATTC | (14) | Confirmation of *fnr3* gene deletion |
| 3R | None | TGAAGTTGGTCAGCAGGC | (14) | |
| 1CodF# | BamHI | TATATAGGATCCTTGCCACATGTCCGCCGG | This study | PCR amplification of the *fnr1* coding region for generation of the *fnr1-3xFlag* construct |
| 1CodR# | Xho I | ATATATCTCGAGGCTGGTGCTGCGGGTCG | This study | |
| 1DownF# | HindIII | TATATAAAGCTTGGGCTGGCCTCATCG | This study | PCR amplification of the downstream region of the *fnr1* gene for generation of the *fnr1-3xFlag* construct |
| 1DownR# | XmaI | ATATATCCCGGGCGCTGATCGGACCCG | This study | |
| 3CodF# | BamHI | TATATAGGATCCCAGCCGACGCCGCCA | This study | |

| | | | | |
|---|---|---|---|---|
| 3CodR# | Xho I | ATATATCTCGAGGGCGCAGGGATCGTTGC | This study | PCR amplification of the *fnr3* coding region for generation of the *fnr3-3xFlag* construct |
| 3DownF# | HindIII | TATATAAAGCTTGACTGCACCGGCCTTGC | This study | PCR amplification of the downstream region of the *fnr3*gene for generation of the *fnr3-3xFlag* construct |
| 3DownR# | XmaI | ATTATCCCGGGCCGCAGCTTGTGAGAGTGG | This study | |
| 1TagF | None | ATTGAAAGCATCAGCCGGCTGATC | This study | Confirmation of 3xFlag insertion into *fnr1* gene |
| 1TagR | None | CAGACCTTGCCCAGGGAACGT | This study | |
| 3TagF | None | GAAATCGGCAACTACCTGGGCC | This study | Confirmation of 3xFlag insertion into *fnr3* gene |
| 3TagR | None | GGCAGGCCCAGATGGTGATCA | This study | |
| 1-3FA | BamHI | GGATCCTTGCCACCATGTCCGCCGGGAACATCGTATG | This study | PCR amplification of the 5` end of the *fnr1* gene to generate the $fnr1^{AR3\text{->}3}$ |
| 1AR3-3FOM | None | CAAGACCTATCAGCACAACCCGAACGGCGGCCAACAGATCACCGG | This study | |
| 1AR3-3ROM | None | CCGGTGATCTGTTGGCCGCCGTTCGGGTTGTGCTGATAGGTCTTG | This study | PCR amplification of the 3` end of the *fnr1* gene to generate the $fnr1^{AR3\text{->}3}$ |
| 3xFlagR | HindIII | AAGCTTCTACTTGTCGTCATCGTCCTTGTAGTCGATGTCGTGG | This study | |
| 3-1FA | XbaI | TCTAGACAGCCGACGCCGCCACAACAAAGAGGAACTCCAACCG | This study | PCR amplification of the 5` end of the *fnr3* gene to generate the $fnr3^{AR3\text{->}1}$ |
| 3AR3-1FOM | None | CAAGACCTTCCAGGAAAATCTCGACGGCGACCGGCAGATCACGGG | This study | |
| 3AR3-1ROM | None | CCCGTGATCTGCCGGTCGCCGTCGAGATTTTCCTGGAAGGTCTTG | This study | Pair with 3xFlagR. Generates 3` end of $fnr3^{AR3\text{->}1}$ |
| P1F | PstI | AGTTTCACTGCAGCGGAAGTTT | This study | PCR amplification of the *fnr1*promoter region to consrtuct the *pfnr1::lacZ* fusion |
| P1R | BglII | AGACCCAAGATCTGGCACAACT | This study | |
| DNAflipP1F | None | ACATGTTTGATAAGTATCATGAAATGCCGT | This study | Pair with P1R. Generates 3` end for *pfnr1*::lacZ* fusion construction |
| DNAflipP1R | None | ACGGCATTTCATGATACTTATCAAACATGT | This study | Pair with P1F. Generates 5` end for *pfnr1*::lacZ* fusion construction |
| TH1F-17 | BamHI | TGGACCGGATCCCATGAACCAGTCTTGC | This study | PCR amplification of *fnr1*gene for cloning into BACTH vectors |
| TH1R-17 | KpnI | TATATAGGTACCCGGCTGGTGCTGCGGGT | This study | |
| TH2F-17 | BamHI | TGGACCGGATCCCATGAGCAAGTCCGAC | This study | PCR amplification of *fnr2*gene for cloning into BACTH vectors |
| TH2R-17 | KpnI | TATGTGGGTACCCGGCCGCAGTTGAATAT | This study | |
| TH3F-17 | BamHI | TGGACCGGATCCCATGTCCAGCCTACCC | This study | PCR amplification of *fnr3*gene for cloning into BACTH vectors |
| TH3R-17 | KpnI | TATATAGGTACCCGGGCGCAGGGATCGTT | This study | |
| Fnr1-I171A-fwd | None | ATCATGAGCCACGAGGCCACCAGCGAACAG | This study | Pair with TH1R-17. Overlapping PCR to generate Fnr1-I171A. |

| | | | | |
|---|---|---|---|---|
| Fnr1-I171A-rev | None | CTGTTCGCTGGTGGCCTCGTGGCTCATGAT | This study | Pair with TH1F-17. Overlapping PCR to generate Fnr1-I171A. |
| Fnr1-E174A-fwd | None | CACGAGATCACCAGCGCACAGAACGTCATCATG | This study | Pair with TH1R-17. Overlapping PCR to generate Fnr1-E174A. |
| Fnr1-E174A-rev | None | CATGATGACGTTCTGTGCGCTGGTGATCTCGTG | This study | Pair with TH1F-17. Overlapping PCR to generate Fnr1-E174A. |
| Fnr3-I156A-fwd | None | ATGAGCCTGGAAGCCTCGCGCGAGCAACGC | This study | Pair with TH3R-17. Overlapping PCR to generate Fnr3-I156A. |
| Fnr3-I156A-rev | None | GCGTTGCTCGCGCGAGGCTTCCAGGCTCAT | This study | Pair with TH3F-17. Overlapping PCR to generate Fnr3-I156A. |
| Fnr3-E159A-fwd | None | GAGCCTGGAAATCTCGCGCGCGCAACGCGTCATG | This study | Pair with TH3R-17. Overlapping PCR to generate Fnr3-E159A. |
| Fnr3-E159A-rev | None | CATGACGCGTTGCGCGCGCGAGATTTCCAGGCTC | This study | Pair with TH3F-17. Overlapping PCR to generate Fnr3-E159A. |
| RT-1 | None | GCCGGCTGATGCTTTCAATGGTCAGGC | This study | 5`RACE to determine transcription start site of *fnr1* gene. Refer to scheme in Figure S6. |
| GSP-1 | None | CGGCAAAACGCTGTTCAGCACGCATG | This study | 5`RACE to determine transcription start site of *fnr1* gene. Refer to scheme in Figure S6. |
| GSP-2 | None | CCGAACAACTGTTCCAGGCGGGCG | This study | 5`RACE to determine transcription start site of *fnr1* gene. Refer to scheme in Figure S6. |

**Supplementary References**


1. Freese,N.H., Norris,D.C. and Loraine,A.E. (2016) Integrated genome browser: Visual analytics platform for genomics. *Bioinformatics*, **32**, 2089–2095.

2. Bordoli,L., Kiefer,F., Arnold,K., Benkert,P., Battey,J. and Schwede,T. (2008) Protein structure homology modeling using SWISS-MODEL workspace. *Nat. Protoc.*, **4**, 1–13.

3. Volbeda,A., Darnault,C., Renoux,O., Nicolet,Y. and Fontecilla-Camps,J.C. (2015) The crystal structure of the global anaerobic transcriptional regulator FNR explains its extremely fine-tuned monomer-dimer equilibrium. *Sci. Adv.*, **1**, e1501086.

4. Souza,E.M., Pedrosa,F.O., Rigo,L.U., Machado,H.B. and Yates,M.G. (2000) Expression of the nifA gene of Herbaspirillum seropedicae: role of the NtrC and NifA binding sites and of the -24/-12 promoter element. *Microbiology*, **146 ( Pt 6**, 1407–18.

5. Simon,R., Priefer,U. and Pühler,A. (1983) A Broad Host Range Mobilization System for In Vivo Genetic Engineering: Transposon Mutagenesis in Gram Negative Bacteria. *Nat. Biotechnol.*, **1**, 784–791.

6. Karimova,G., Gauliard,E., Davi,M., Ouellette,S.P. and Ladant,D. (2017) Protein–Protein Interaction: Bacterial Two-Hybrid. In Journet,L., Cascales,E. (eds), *Bacterial Protein Secretion Systems: Methods and Protocols*. Springer New York, New York, NY, pp. 159–176.

7. Schäfer,A., Tauch,A., Jäger,W., Kalinowski,J., Thierbach,G. and Pühler,A. (1994) Small mobilizable multi-purpose cloning vectors derived from the Escherichia coli plasmids pK18 and pK19: Selection of defined deletions in the chromosome of Corynebacterium glutamicum. *Gene*, **145**, 69–73.

8. Quandt,J. and Hynes,M.F. (1993) Versatile suicide vectors which allow direct selection for gene replacement in gram-negative bacteria. *Gene*, **127**, 15–21.

9. Woodley,P., Buck,M. and Kennedy,C. (1996) Identification of sequences important for recognition of vnf genes by the VnfA transcriptional activator in Azotobacter vinelandii. *FEMS Microbiol. Lett.*, **135**, 213–221.

10. Karimova,G., Ullmann,A. and Ladant,D. (2001) Protein-protein interaction between Bacillus stearothermophilus tyrosyl-tRNA synthetase subdomains revealed by a

bacterial two-hybrid system. *J. Mol. Microbiol. Biotechnol.*, **3**, 73–82.

11. Karimova,G., Pidoux,J., Ullmann,A. and Ladant,D. (1998) A bacterial two-hybrid system based on a reconstituted signal transduction pathway. *Proc. Natl. Acad. Sci. U. S. A.*, **95**, 5752–6.

12. Karimova,G., Dautin,N. and Ladant,D. (2005) Interaction network among Escherichia coli membrane proteins involved in cell division as revealed by bacterial two-hybrid analysis. *J. Bacteriol.*, **187**, 2233–43.

13. Alting-Mees,M.A. and Short,J.M. (1989) pBluescript II: gene mapping vectors. *Nucleic Acids Res.*, **17**, 9494.

14. Batista,M.B., Sfeir,M.Z.T., Faoro,H., Wassem,R., Steffens,M.B.R., Pedrosa,F.O., Souza,E.M., Dixon,R. and Monteiro,R. a (2013) The Herbaspirillum seropedicae SmR1 Fnr orthologs controls the cytochrome composition of the electron transport chain. *Sci. Rep.*, **3**, 2544.
